# Slowing down x-ray photons in a vibrating recoilless resonant absorber

**DOI:** 10.1038/s41598-022-24114-8

**Published:** 2022-11-24

**Authors:** I. R. Khairulin, Y. V. Radeonychev, Olga Kocharovskaya

**Affiliations:** 1 Institute of Applied Physics of the Russian Academy of Sciences, 46 Ulyanov Street, Nizhny Novgorod, 603950 Russia; 2 Department of Physics and Astronomy and Institute for Quantum Science and Engineering, Texas A&M University, College Station, TX 77843-4242 USA

**Keywords:** Single photons and quantum effects, Slow light, X-rays

## Abstract

Recently, an observation of acoustically induced transparency (AIT) of a stainless-steel foil for resonant 14.4-keV photons from a radioactive ^57^Co Mössbauer source due to collective uniform oscillations of atomic nuclei was reported [Phys Rev Lett 124,163602, 2020]. In this paper, we propose to use the steep resonant dispersion of the absorber within the AIT spectral window to dramatically reduce a propagation velocity of γ-ray and x-ray photons. In particular, we show that a significant fraction (more than 40%) of a 97-ns γ-ray single-photon wave packet from a ^57^Co radioactive source can be slowed down up to 3 m/s and delayed by 144 ns in a ^57^Fe-enriched stainless-steel foil at room temperature. We also show that a similarly significant slowing down up to 24 m/s and a delay by 42 ns can be achieved for more than 70% of the 100-ns 14.4-keV x-ray single-photon pulse from a synchrotron Mössbauer source available at European Synchrotron Radiation Facility (ESRF) and Spring-8 facility. The propagation velocity can be widely controlled by changing the absorber vibration frequency. Achieving the propagation velocity on the order of 1–50 m/s would set a record in the hard x-ray range, comparable to what was obtained in the optical range.

## Introduction

Controlled single-photon delay lines are in high demand for storage, long-distance communication and processing of quantum information. Recently, a large number of techniques to delay optical pulses, based on slowing down pulse propagation in the medium, were proposed (see^1–16^ and references therein). Most of them utilize temporal^1–11^ or spatial^12–16^ dispersion of the medium and are based on producing a sharp dependence of the refraction index on the frequency or wavevector within the transparency spectral window covering the band of propagating light. Electromagnetically induced transparency (EIT) is one of the prevailing paradigms in the field^1–5,17,18^. The transparency induced via coherent population oscillation^6,7^, Autler–Townes splitting (ATS)^10,11^, opto-mechanical induced transparency^8,9^, transparency induced in structured optical waveguides with coupled micro-cavities^13,14^ and in other metadevices^15,16^ are conceptually related to EIT.

EIT made it possible to achieve the record slow-light velocity of about 17 m/s in a nanokelvin gas of sodium atoms^2^, 90 m/s in an optically dense hot (≈360 K) rubidium gas^4^, and 45 m/s in an optically dense crystal of Pr:Y_2_SiO_5_ at a temperature of 5 K^5^. The observation of a 13 ms delay in the polarization rotation of light transmitted through a ^87^Rb cell at room temperature can be interpreted as a group velocity of 8 m/s^3^. Slowing down light to 57.5 m/s in a ruby crystal at room temperature was observed via transparency induced due to coherent population oscillation^6^. All these impressive results were obtained in the optical range.

The recoilless resonant interaction of high-energy x-ray photons with atomic nuclei is similar to the interaction of optical photons with atomic electrons. At the same time, it has some attractive features making it promising for development of very compact quantum photonic devices in the x-ray range. Hard x-ray photons can be more easily and reliably detected than optical photons and focused to nano-size spot^19^, as well as penetrate through many optically opaque materials. The synchrotron Mössbauer sources (SMS)^20–24^ and ^57^Co radioactive Mössbauer sources (RMS)^25–31^ can produce narrowband (on the order of megahertz) heralded 14.4-keV photons with a coherence length of about 40 m. Spectrally narrow x-ray pulses can also be produced from synchrotron radiation (SR) with use of a high-speed mechanical chopper^32^ or polarization filtering^33^. The respective 14.4-keV resonant recoilless (Mössbauer) nuclear transitions in ^57^Fe nuclide normally have a few orders of magnitude narrower linewidths at room temperature (several megahertz) than transitions of bound electrons in atoms (several gigahertz), corresponding to very high Q-factor on the order of 10^12^. Together with the high density of the nuclei in a solid (up to 10^23^ cm^-3^) this allows for a large resonant optical depth with a small physical length of the absorber (an *e*-fold attenuation of 14.4-keV radiation is achieved in a ^57^Fe foil of about 70 nm thickness). These properties of the recoilless photons and nuclei are promising for the development of very compact quantum photonic devices in the x-ray range.

However, most approaches well suited for optical and infrared ranges are currently not feasible for hard x-ray radiation with a wavelength shorter than an angstrom. This is due to the fact that high quality cavities, sufficiently high-intensity narrowband driving lasers, radiation pulses with controllable pulse shape and duration, as well as dielectric spatial structures with a characteristic scale on the order of the radiation wavelength, which are widely used in the optical range, are currently not available in the x-ray range.

At the same time, a number of alternative efficient methods for controlling the resonant interaction of x-ray photons with nuclear ensembles have been developed (see^26–47^ and references therein). In particular, several techniques have been proposed and implemented to reduce resonant nuclear absorption accompanied by a decrease in the propagation velocity of x-ray photons^33,34,36–38^. The 25% reduction in absorption of 14.4-keV photons was observed via anti-crossing of the upper energy sublevels of ^57^Fe nuclei in a crystal of FeCO_3_, which took place at 30 K due to the temperature-induced hyperfine magnetic field adjustment^34^. The estimated group velocity was on the order of 10^3^ m/s. The decelerated propagation of 14.4-keV photons from a ^57^Co-source with velocity down to 660 m/s at room temperature through a ^57^Fe absorber due to a steep dispersion accompanying a quadrupole doublet structure of the 14.4-keV transition inherent to some compounds was observed in^39^. The 4.5-fold suppression of 14.4-keV collective coherent emission in the given direction from two ^57^Fe layers imbedded into a specifically designed planar cavity was reported in^38^ with a perspective to achieve the 15-fold absorption suppression in the ideal case of the single-line ^57^Fe absorber. Subsequently, the subluminal propagation with controlled velocity of spectrally narrow 14.4-keV x-ray pulses through a nano-sandwich x-ray cavity with a single ^57^Fe layer in the reflection geometry was observed in^33^. The photon group velocity estimated from the measured controlled delay of the reflected pulses up to 35 ns was less than 3 × 10^4^ m/s at room temperature. Recently, 148-fold suppression of the resonant absorption of the 14.4-keV photons from ^57^Co source in the ^57^Fe absorber was achieved via acoustically induced transparency (AIT) due to collective uniform oscillations of atomic nuclei^37^. The estimated photon group velocity was 5.6 × 10^3^ m/s.

In this paper, we propose a technique for dramatic slowing down the x-ray photons up to several meters per second at room temperature, which is based on a sharp dispersion accompanying the AIT spectral window. The possibility of decelerating a significant part of the 14.4-keV single-photon wave packet from both RMS and SMS in a ^57^Fe-enriched stainless-steel foil under currently available experimental conditions is predicted.

The paper is organized as follows. In section "Basic idea" we analyze the dispersive properties of a vibrating medium in the laboratory reference frame considering as an example the ^57^Fe nuclear absorber. We show how a spectral dip in resonant nuclear absorption, accompanied by a steep normal dispersion, appears due to vibration of the absorber leading to a dramatic decrease in the group velocity of a narrow-band wave packet and the propagation velocity of a broadband wave packet. In section "Group velocity in vibrating reference frame", we provide a complementary physical picture of the decrease in the group and propagation velocities using the reference frame co-moving with the vibrated absorber. In section "Photon waveform in the vibrating absorber", we derive the integral equation for intensity of the transmitted single-photon wave packet proportional to the time dependence of the photon detection probability (the photon waveform). In the next three sections "Slowing down Gaussian photons", "Slowing down Lorentzian photons", "Slowing down Lorentz-squared photons", this integral equation is used to determine the propagation delay and propagation velocity of the single-photon wave packet for various spectral shapes, namely, Gaussian, Lorentzian and Lorentz-squared. In sections "Slowing down Lorentzian photons" and "Slowing down Lorentz-squared photons", we also determine the optimal values of both the vibration frequency and the optical depth of the absorber for observing the longest propagation delay and slowest propagation velocity of 14.4-keV photons emitted by the ^57^Co radioactive source and the ESRF synchrotron source in a stainless-steel foil enriched with ^57^Fe nuclide. In section "Conclusion", we summarize the results.

## Basic idea

As is well known, the propagation of a quasi-monochromatic wave packet

1 $$E(t,z) = A(t,z)e^{{ - i\left( {\omega_{0} {\kern 1pt} t - k_{0} {\kern 1pt} z} \right)}} ,$$

(where $\left| {{{dA} \mathord{\left/ {\vphantom {{dA} {dt}}} \right. \kern-\nulldelimiterspace} {dt}}} \right| < < \omega_{0} \left| A \right|$, $\left| {{{dA} \mathord{\left/ {\vphantom {{dA} {dz}}} \right. \kern-\nulldelimiterspace} {dz}}} \right| < < k_{0} \left| A \right|$) through a transparent medium of the length *L*, $0 \le z \le L$, can be characterized by the group velocity, $v_{g} \equiv \left. {\left( {{{d\omega } \mathord{\left/ {\vphantom {{d\omega } {dk^{\prime}}}} \right. \kern-\nulldelimiterspace} {dk^{\prime}}}} \right)} \right|_{{\omega = \omega_{0} }}$, subject to $v_{g}$ does not depend on $\omega$. Here $\omega$ and $k(\omega ) = k^{\prime}(\omega ) + ik^{\prime\prime}(\omega )$, where $k^{\prime\prime} \ll k^{\prime}$, are the frequency and complex wavenumber of the wave packet Fourier constituent,

2 $$\overline{E}(\omega ,z) = \frac{1}{2\pi }\int\limits_{ - \infty }^{\infty } {E(t,z)e^{i\omega t} dt} ,$$

which can be written in the form $\overline{E}(\omega ,z) = \overline{E}(\omega )\exp \left[ {ik(\omega )z} \right]$. In the case of a transparent medium, the group velocity of the wave packet is determined by the frequency dispersion of the medium, $\omega \sqrt {\varepsilon^{\prime}(\omega )} \simeq ck^{\prime}(\omega )$ (where $c$ is the speed of light in vacuum and $\varepsilon = \varepsilon^{\prime} + i\varepsilon^{\prime\prime}$ ($\varepsilon^{\prime\prime} \ll \varepsilon^{\prime}$) is the medium dielectric permittivity at frequency $\omega$),

3 $$v_{g} = \frac{c}{{\sqrt {\varepsilon^{\prime}(\omega_{0} )} + \frac{{\omega_{0} }}{{2\sqrt {\varepsilon^{\prime}(\omega_{0} )} }} \cdot \frac{{d\varepsilon^{\prime}(\omega_{0} )}}{d\omega }}}.$$

As follows from Eq. (3), achieving a low group velocity implies a presence of a normal (${{d\varepsilon ^{\prime}} \mathord{\left/ {\vphantom {{d\varepsilon ^{\prime}} {d\omega }}} \right. \kern-\nulldelimiterspace} {d\omega }}$ > 0) and steep (large derivative ${{d\varepsilon ^{\prime}} \mathord{\left/ {\vphantom {{d\varepsilon ^{\prime}} {d\omega }}} \right. \kern-\nulldelimiterspace} {d\omega }}$) dispersion in a transparent medium. If a narrow dip is created within the absorption spectrum of a resonant quantum transition (spectral transparency window), then it is always accompanied by normal and steep dispersion, described by the Kramers–Kronig relation. For this reason, various techniques for achieving slow group velocity differ from each other primarily by the physical mechanism that provides the transparency window. For example, driving a resonant transition in a three-level medium by a sufficiently strong field can lead to transparency for the field resonant to an adjacent atomic transition due to interference of the induced atomic transitions (EIT) or, in the case of a significantly stronger field, due to the disappearance of the atom–field interaction as a result of Autler–Townes splitting. Both mechanisms cause light slowing^2,17,18^.

**Figure 1:**
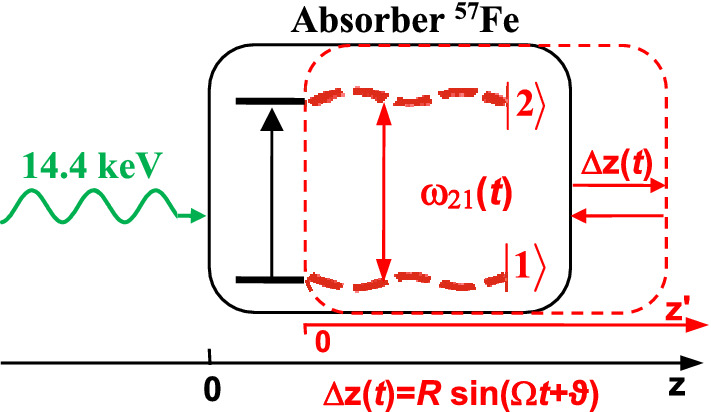
(Color online) Energy scheme of 14.4-keV photon propagation through the vibrating absorber used in the experiment^37^. Recoilless 14.4-keV photons (*λ*_0_ = 2π*c*/*ω*_0_≈0.86 Å) resonantly interact with transition $\left| 1 \right\rangle \leftrightarrow \left| 2 \right\rangle$ of ^57^Fe nuclei when propagating through the single-line ^57^Fe absorber. They are resonantly absorbed in motionless absorber (black lines). Harmonic vibration of the absorber as a whole (piston-like vibration) with circular frequency Ω, amplitude *R*, and initial phase *ϑ* along the photon propagation direction (marked in red) leads to periodic temporary variation in $\left| 1 \right\rangle \leftrightarrow \left| 2 \right\rangle$ transition frequency ω_21_(*t*) (dashed red curves) due to the Doppler effect. It modifies the interaction of photon with absorber and can result in AIT (see Fig. 2 and text). The axis *z* labels the laboratory reference frame, red axis *z*' labels reference frame of the vibrating absorber, and Δ*z* = *z−z*'.

Similarly, piston-like vibration of a resonant two-level absorber along the direction of photon propagation can result in AIT^37,46^, which should also lead to a decrease in the group velocity of photons. In order to show this, let us calculate the susceptibility of the vibrating medium in the laboratory reference frame. For this purpose, following the references^37,46^, we find the resonant nuclear polarization $P_{21}$ of quantum transition $\left| 1 \right\rangle \to \left| 2 \right\rangle$ induced under the action of a monochromatic Fourier constituent Eq. (2) of the field Eq. (1) at the entrance of the absorber, $z = 0$. The two-level absorber with quantum transition frequency *ω*_21_ sinusoidally vibrates as a whole (piston-like) along *z*-axis (Fig. 1),

4 $$z = z^{\prime} + R\sin (\Omega t + \vartheta ),$$

where *z*′ is the coordinate in the vibrating reference frame, Ω, *R*, and *ϑ* are the circular frequency, amplitude, and initial phase of vibration, respectively. Equation (4) describes the case when the thickness of the absorber, *L*, is much less than the wavelength of sound, $L \ll 2\pi V_{sound} /\Omega$ (where *V*_*sound*_ is the speed of sound in the absorber) and its motion is non-relativistic,$R\Omega \ll c$.

The induced resonant polarization in the nuclear absorber can be represented within the model of electric-dipole field-matter interaction (see^26,30,31,37,46^ and references therein): $P_{21} = f_{a} Nd_{12} \rho_{21}$, where *ρ*_21_ is the induced coherence of the resonant nuclear transition, *f*_*a*_ is the Lamb-Mössbauer factor accounting for the probability of recoilless absorption, *N* is the concentration of resonant nuclei, and $d_{12} = d_{21}^{*}$ is the effective dipole moment of the resonant nuclear transition $\left| 1 \right\rangle \leftrightarrow \left| 2 \right\rangle$. With an effective dipole moment, this model correctly describes the magnetic-dipole interaction of 14.4-keV photons with ^57^Fe nuclei^25,26,30^. Motion of the absorber leads to shifting its quantum transition frequency, *ω*_21_, relative to the spectral line of the motionless source due to the Doppler effect. The respective transition frequency of the moving absorber is $\tilde{\omega }_{21} = \omega_{21} + k_{0} {\kern 1pt} v$, where *v* = *dz*/*dt* is the velocity of the absorber in the laboratory reference frame. As a result, the master equation for coherence *ρ*_21_ induced by the monochromatic Fourier constituent of the incident field Eq. (1), has the form

5 $$\frac{{\partial \rho_{21} }}{\partial t} + i\left[ {\omega_{21} + k_{0} R\Omega \cos (\Omega t + \vartheta )} \right]\rho_{21} + \gamma_{21} \rho_{21} = \frac{i}{\hbar }n_{12} d_{21} \overline{E}(\omega ,z)e^{ - i\omega t} ,$$

where *n*_12_ = *ρ*_11_ − *ρ*_22_ is the population difference between the states $\left| 1 \right\rangle$ and $\left| 2 \right\rangle$ and *γ*_21_ is the half-width at half-maximum (HWHM) of spectral line of the resonant transition, and the relation $\Omega \ll \omega_{21} ,\omega$ is assumed. Since the considered x-ray field is too weak to change the populations of the states $\left| 1 \right\rangle$ and $\left| 2 \right\rangle$, we assume below that $n_{12} = 1$. Solution of Eq. (5) can be searched in the form

6 $$\rho_{21} = \sigma_{21} e^{{ - ik_{0} R\sin (\Omega t + \vartheta )}} e^{ - i\omega t} .$$

Then Eq. (5) for the amplitude of coherence, *σ*_21_, reads as

7 $$\frac{{\partial \sigma_{21} }}{\partial t} + i\left( {\omega_{21} - \omega } \right)\sigma_{21} + \gamma_{21} \sigma_{21} = \frac{i}{\hbar }n_{12} d_{21} \overline{E}\left( {\omega ,z} \right)e^{ip\sin (\Omega t + \vartheta )} ,$$

where $p = k_{0} R$ is the modulation index of the absorber’s quantum transition frequency due to its vibration. Using the Jacobi-Anger expansion, $e^{ \pm ip\sin \phi } = \sum\limits_{n = - \infty }^{\infty } {J_{n} (p)e^{ \pm in\phi } }$ (where *J*_*n*_(*p*) is the *n*-th Bessel function of the first kind) one can find from Eq. (7) the amplitude of the induced resonant coherence *σ*_21_ as

8 $$\sigma_{21} = \frac{i}{{\hbar \gamma_{21} }}n_{12} d_{21} \overline{E}\left( {\omega ,z} \right)\sum\limits_{n = - \infty }^{\infty } {\eta_{n} J_{n} (p)e^{in(\Omega t + \vartheta )} } ,$$

where

9 $$\eta_{n} = \frac{1}{{1 + i{{\left( {\omega_{21} - \omega + n\Omega } \right)} \mathord{\left/ {\vphantom {{\left( {\omega_{21} - \omega + n\Omega } \right)} {\gamma_{21} }}} \right. \kern-\nulldelimiterspace} {\gamma_{21} }}}}.$$

So, according to Eqs. (6) and (8), the monochromatic incident field $\overline{E}(\omega ,z)e^{ - i\omega t}$ induces nuclear coherence not only at the frequency of the field, $\omega$, but also at the series of frequencies shifted by a multiple of the vibration frequency, $\omega + q\Omega$, $q \in {\mathbb{Z}},\;q \ne 0$:

10 $$\rho_{21} = \rho_{21}^{\left( 0 \right)} + \sum\limits_{\begin{subarray}{l} q = - \infty \\ \,\,q \ne 0 \end{subarray} }^{\infty } {\rho_{21}^{\left( q \right)} } ,$$

where

11 $$\rho_{21}^{\left( 0 \right)} = in_{12} e^{ - i\omega \;t} \frac{{d_{21} \overline{E}\left( {\omega ,z} \right)}}{{\hbar \gamma_{21} }}\sum\limits_{n = - \infty }^{\infty } {\eta_{n} J_{n}^{2} (p)}$$

is the nuclear coherence induced at the frequency of the monochromatic field, $\omega$, and

12 $$\rho_{21}^{\left( q \right)} = in_{12} e^{{ - i\left( {\omega + q\Omega } \right)t - iq\vartheta }} \frac{{d_{21} \overline{E}\left( {\omega ,z} \right)}}{{\hbar \gamma_{21} }}\sum\limits_{n = - \infty }^{\infty } {\eta_{n} J_{n} (p)J_{n + q} (p)} ,\quad {\text{for}}\;q \ne 0,$$

is the nuclear coherence induced at the combination frequency, $\omega_{q} = \omega + q\Omega$ due to the absorber vibration.

According to Eqs. (11), (12), the polarization with a comb-like spectrum transforms the incident monochromatic field into a multi-frequency field inside the absorber. The amplitudes of the central spectral component and sidebands of the nuclear response are determined by the modulation index $p$. As can be seen from Eqs. (11), (12) and was shown in^31,40–42,47^, at some values of the modulation index (for example, at $p = 1.84$), the amplitudes of the central component and several sidebands of the nuclear response are comparable. This makes it possible to transform the incident monochromatic or quasi-monochromatic radiation Eq. (1) into a sequence of ultrashort pulses, which was theoretically studied in^40,41,47^ and experimentally implemented in^31,42^.

The case of AIT with preserving spectral-temporal characteristics of γ-ray photons was realized in^37^ at other values of the modulation index (see also^46^), namely at

13 $$p = p_{i} ,\;{\text{where}}\;p_{1} \approx 2.4,\,\,\,p_{2} \approx 5.5,\,\,\,\,p_{3} \approx 8.6, \ldots ,$$

corresponding to the amplitude of the absorber vibration

14 $$R = R_{i} ,\,\,{\text{where}}\,\,\,\,R_{1} \approx 0.38\lambda ,\,\,\,R_{2} \approx 0.88\lambda ,\,\,\,\,R_{3} \approx 1.37\lambda , \ldots$$

For these values, $J_{0} (p_{i} ) = 0$ in Eqs. (11) and (12). If the absorber vibration frequency is large enough, $\Omega \gg \gamma_{21}$, then in the case of the near-resonant monochromatic field, $\left| {\omega_{21} - \omega } \right| \ll \Omega$, all other terms in sums of Eqs. (11) and (12) are negligible since $\eta_{n} \approx \left( {\frac{{\gamma_{21} }}{n\Omega }} \right)^{2} - i\frac{{\gamma_{21} }}{n\Omega }$, $n \ne 0$ (see also supplemental material in^37^). In other words, the nuclear response both at the frequency of the incident monochromatic field, $\omega$, (Eq. (11)) and at the combination frequencies, $\omega_{q}$, (Eq. (12)) is vanishing, i.e. the medium becomes transparent, preserving the spectral-temporal characteristics of the field.

Now consider the dependence of the nuclear response at the frequency of the incident monochromatic field, $\omega$, Eq. (11), for an arbitrary absorber vibration frequency, $\Omega$, and for a certain vibration amplitude, $R = R_{1}$, corresponding to the modulation index $p = p_{1}$ (the nuclear response at the combination frequencies, Eq. (12), will be considered afterwards). In this case, the nuclear susceptibility, $\chi_{21} (\omega )$, follows from Eq. (11) according to the relation $P_{21} = f_{a} Nd_{12} \rho_{21}^{(0)} = \chi_{21} (\omega )\overline{E}(\omega ,z)e^{ - i\omega t}$ and reads as

15 $$\chi_{21} \left( \omega \right) = \chi_{0} \sum\limits_{n = - \infty }^{\infty } {J_{n}^{2} \left( {p_{1} } \right)\frac{{{{\left( {\omega_{21} - \omega + n\Omega } \right)} \mathord{\left/ {\vphantom {{\left( {\omega_{21} - \omega + n\Omega } \right)} {\gamma_{21} }}} \right. \kern-\nulldelimiterspace} {\gamma_{21} }} + i}}{{{{\left( {\omega_{21} - \omega + n\Omega } \right)^{2} } \mathord{\left/ {\vphantom {{\left( {\omega_{21} - \omega + n\Omega } \right)^{2} } {\gamma_{21}^{2} }}} \right. \kern-\nulldelimiterspace} {\gamma_{21}^{2} }} + 1}}} ,\quad \chi_{0} = \frac{{f_{a} Nn_{12} \left| {d_{21} } \right|^{2} }}{{\hbar \gamma_{21} }}.$$

**Figure 2:**
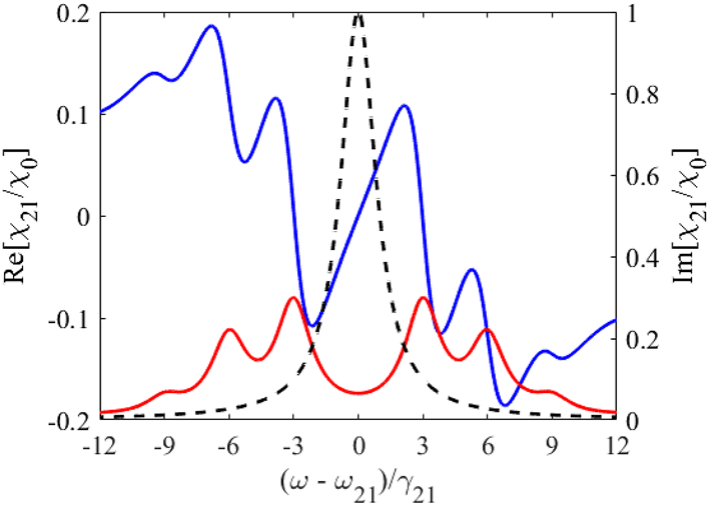
(Color online) Absorption (red curve, right axis) and dispersion (blue curve, left axis) of the vibrating resonant absorber at the frequency of the monochromatic field, $\omega$, according to Eq. (15) in the laboratory reference frame for $p = p_{1} = 2.4$ and ${\Omega \mathord{\left/ {\vphantom {\Omega {\gamma_{21} }}} \right. \kern-\nulldelimiterspace} {\gamma_{21} }} = 3$. The black dashed curve (right axis) is the absorption line of the motionless absorber plotted according to Eq. (15) for $p_{1} = 0$. The black dashed curve here also represents the incident wave packet with Lorentz spectrum of the HWHM $\Delta = \gamma_{21}$.

According to Eq. (15), the hilly-like response spectrum of the vibrating absorber at the frequency of the monochromatic field, $\omega$, (Fig. 2) is the result of a weighted sum of Lorentzian contours separated by the vibration frequency, $\Omega$. The imaginary part of the nuclear susceptibility, Eq. (15), has a dip centered at the nuclear transition frequency $\omega_{21}$ between two absorption peaks shifted at the absorber vibration frequency, $\pm \Omega$, forming the AIT spectral window (Fig. 2, red line). The depth and width of the AIT spectral window is determined by the ratio between the absorber vibration frequency, $\Omega$, and the absorber transition halfwidth, $\gamma_{21}$, similar to the transparency window induced due to Autler-Townes splitting by a strong driving field with frequency $\Omega$^48^. At large vibration frequency, $\Omega \gg \gamma_{21}$, the width of the AIT spectral window tends to $2\Omega$ and is much larger than the nuclear transition linewidth. In this case, the AIT is nearly perfect, ${\text{Im}} \left\{ {\chi_{21} \left( \omega \right)} \right\} \approx 2\chi_{0} \sum\limits_{n = 1}^{\infty } {J_{n}^{2} \left( {p_{1} } \right){{\gamma_{21}^{2} } \mathord{\left/ {\vphantom {{\gamma_{21}^{2} } {(n\Omega )^{2} }}} \right. \kern-\nulldelimiterspace} {(n\Omega )^{2} }}} \ll \chi_{0}$.

If the absorber vibration frequency is on the order of the nuclear transition linewidth, $\Omega \sim \gamma_{21}$, the AIT window becomes narrower than $2\Omega$ (Fig. 2, red line), whereas the resonant nuclear absorption (the imaginary part of the absorber susceptibility at $\omega = \omega_{21}$ in Eq. (15)) increases and strongly depends on the absorber vibration frequency. For this reason, it is convenient to introduce an effective resonant optical depth (effective Mössbauer thickness) of the vibrating absorber,

16 $$T_{a}^{{\left( {eff} \right)}} \equiv T_{a}^{{\left( {eff} \right)}} (\omega_{21} ) = {{4\pi \omega_{21} {\text{Im}} \left[ {\chi_{21} \left( {\omega_{21} } \right)} \right]L} \mathord{\left/ {\vphantom {{4\pi \omega_{21} {\text{Im}} \left[ {\chi_{21} \left( {\omega_{21} } \right)} \right]L} c}} \right. \kern-\nulldelimiterspace} c},$$

generalizing the usual resonant optical depth (Mössbauer thickness) of the motionless absorber,

17 $$T_{a} = \frac{{4\pi \omega_{21} L}}{c}\chi_{0} .$$

According to Eqs. (15), (16), the effective resonant optical depth can be expressed as

18 $$T_{a}^{{\left( {eff} \right)}} = T_{a} \sum\limits_{n = - \infty }^{\infty } {\frac{{J_{n}^{2} \left( {p_{1} } \right)}}{{\left( {{{n\Omega } \mathord{\left/ {\vphantom {{n\Omega } {\gamma_{21} }}} \right. \kern-\nulldelimiterspace} {\gamma_{21} }}} \right)^{2} + 1}}} = \left\{ \begin{gathered} T_{a} ,\quad \Omega = 0; \hfill \\ \frac{{T_{a} \gamma_{21}^{2} }}{{\Omega^{2} }}\sum\limits_{\begin{subarray}{l} n = - \infty \\ \,\,n \ne 0 \end{subarray} }^{\infty } {\frac{{J_{n}^{2} \left( {p_{1} } \right)}}{{n^{2} }}} \ll T_{a} , \, \Omega \gg \gamma_{21} . \hfill \\ \end{gathered} \right.$$

As shown in Fig. 2 by blue line, the dispersion of the nuclear resonant transition (the real part of susceptibility in Eq. (15)) is normal and almost linearly depends on the frequency with a steep slope over most of the AIT spectral window. In the case of a sufficiently small absorption of the field inside the AIT window, $T_{a}^{{\left( {eff} \right)}} \ll 1$, the slope of the dispersion curve determines the group velocity of a *narrowband* wave packet, the spectral width of which is much less than the width of the AIT window. The corresponding condition is $\Delta \ll \Omega$, where $\Delta$ is the HWHM of the field spectrum. In the case of a large absorber vibration frequency, $\Omega \gg \gamma_{21}$, the narrowband wave packet can be essentially broader than the absorber linewidth, $\gamma_{21} \ll \Delta \ll \Omega$. In the case $\Omega \sim \gamma_{21}$, the narrowband wave packet should meet the condition $\Delta \ll \gamma_{21}$.

The group velocity of the narrowband wave packet resonant to the nuclear transition, $\omega_{0} = \omega_{21}$, follows from Eqs. (3) and (15) with accounting for $\varepsilon \left( \omega \right) = 1 + 4\pi \chi_{21} \left( \omega \right)$,

19 $$v_{g} = \frac{c}{{1 + c\frac{{f_{a} Nn_{12} }}{{2\gamma_{21} }}\sigma_{0} \sum\limits_{n = - \infty }^{\infty } {J_{n}^{2} \left( {p_{1} } \right)\frac{{\left( {{{n\Omega } \mathord{\left/ {\vphantom {{n\Omega } {\gamma_{21} }}} \right. \kern-\nulldelimiterspace} {\gamma_{21} }}} \right)^{2} - 1}}{{\left\{ {\left( {{{n\Omega } \mathord{\left/ {\vphantom {{n\Omega } {\gamma_{21} }}} \right. \kern-\nulldelimiterspace} {\gamma_{21} }}} \right)^{2} + 1} \right\}^{2} }}} }},$$

where ${{\sigma_{0} = 4\pi \omega_{21} d_{21}^{2} } \mathord{\left/ {\vphantom {{\sigma_{0} = 4\pi \omega_{21} d_{21}^{2} } {(c\hbar \gamma_{21} )}}} \right. \kern-\nulldelimiterspace} {(c\hbar \gamma_{21} )}} = 2.56 \times 10^{ - 18} {\text{cm}}^{{2}}$ is the cross-section of the resonant 14.4-keV transition of ^57^Fe nucleus, assumed to be naturally broadened, ${{\gamma_{21} } \mathord{\left/ {\vphantom {{\gamma_{21} } {(2\pi )}}} \right. \kern-\nulldelimiterspace} {(2\pi )}} = 0.56{\text{ MHz}}$. Below we consider the absorber in the form of a stainless-steel foil at room temperature. In this case, the typical value of the Lamb-Mössbauer factor is $f_{a} = 0.75$.

As follows from Eq. (19), the group velocity of the narrowband wave packet does not depend on either the optical depth or physical thickness (see also Fig. 4d below), but only on the nuclear parameters and the concentration of nuclei. The minimum group velocity is achieved at maximum concentration of ^57^Fe nuclei, $N$. Therefore, we consider hereinafter a stainless-steel foil Fe_70_Cr_19_Ni_11_, with 95% of ^57^Fe in iron fraction^30,37,49,50^ corresponding to $N \approx 5.5 \times 10^{22} {\text{cm}}^{{ - 3}}$. In this case, the term before the sum in Eq. (19) equals 4.5 × 10^8^. According to Eq. (18), the transparency condition $T_{a}^{{\left( {eff} \right)}} \ll 1$ limits the absorber vibration frequency in an optically deep absorber, $T_{a} > 1$, to approximately $\Omega \ge 3\gamma_{21}$. Thus, for $\Omega = 3\gamma_{21}$, the group velocity of a narrowband wave packet, $\Delta \ll \Omega ,\gamma_{21}$ is $v_{g} \approx 12\,{{\text{.4 m}} \mathord{\left/ {\vphantom {{\text{.4 m}} {\text{s}}}} \right. \kern-\nulldelimiterspace} {\text{s}}}$. Increase in the frequency of the absorber vibration leads to increase in the group velocity. However, the group velocity remains significantly less than the speed of light, approaching the latter only at ${\Omega \mathord{\left/ {\vphantom {\Omega {\gamma_{21} }}} \right. \kern-\nulldelimiterspace} {\gamma_{21} }} > 10^{4}$.

The group velocity of propagation of the narrowband wave packet through the absorber of thickness $L$ can also be characterized by the group delay, $\tau_{g}$, at the exit from the absorber relative to the transmission time, ${L \mathord{\left/ {\vphantom {L c}} \right. \kern-\nulldelimiterspace} c}$, in free space,

20 $$v_{g} = \frac{L}{{\tau_{g} + {L \mathord{\left/ {\vphantom {L c}} \right. \kern-\nulldelimiterspace} c}}}\mathop \simeq \limits^{{\tau_{g} \gg {L \mathord{\left/ {\vphantom {L c}} \right. \kern-\nulldelimiterspace} c}}} {L \mathord{\left/ {\vphantom {L {\tau_{g} }}} \right. \kern-\nulldelimiterspace} {\tau_{g} }}.$$

Following Eqs. (18)–(20), the group delay can be estimated as

21 $$\tau_{g} = \frac{{T_{a} }}{{2\gamma_{21} }}\sum\limits_{n = - \infty }^{\infty } {J_{n}^{2} \left( {p_{1} } \right)\frac{{\left( {{{n\Omega } \mathord{\left/ {\vphantom {{n\Omega } {\gamma_{21} }}} \right. \kern-\nulldelimiterspace} {\gamma_{21} }}} \right)^{2} - 1}}{{\left[ {\left( {{{n\Omega } \mathord{\left/ {\vphantom {{n\Omega } {\gamma_{21} }}} \right. \kern-\nulldelimiterspace} {\gamma_{21} }}} \right)^{2} + 1} \right]^{2} }}} < \frac{{T_{a}^{{\left( {eff} \right)}} }}{{2\gamma_{21} }} \, \to ^{{\Omega \gg \gamma_{21} }} \, \frac{{T_{a}^{{\left( {eff} \right)}} }}{{2\gamma_{21} }}.$$

As follows from Eq. (21), due to the AIT condition $T_{a}^{{\left( {eff} \right)}} \ll 1$, the group delay of the narrowband wave packet cannot exceed the decay time of the resonant nuclear transition, namely, $\tau_{g} \ll \left( {2\gamma_{21} } \right)^{ - 1}$.

Now let us consider the nuclear response to the incident monochromatic field at the combination frequencies, $\omega_{q} = \omega + q\Omega$, $q \in {\mathbb{Z}},\;q \ne 0$, Eq. (12). The nuclear susceptibility $\chi_{21}^{(q)} \left( \omega \right)$ at the combination frequency $\omega_{q}$ follows from Eq. (12) according to the relation $P_{21}^{(q)} = f_{a} Nd_{12} \rho_{21}^{(q)} = \chi_{21}^{q} (\omega )\overline{E}(\omega ,z)e^{{ - i\omega_{q} t}}$ and reads as

22 $$\chi_{21}^{\left( q \right)} \left( \omega \right) = \chi_{0} e^{ - iq\vartheta } \sum\limits_{n = - \infty }^{\infty } {J_{n} (p_{1} )J_{n + q} (p_{1} )\frac{{{{\left( {\omega_{21} - \omega + n\Omega } \right)} \mathord{\left/ {\vphantom {{\left( {\omega_{21} - \omega + n\Omega } \right)} {\gamma_{21} }}} \right. \kern-\nulldelimiterspace} {\gamma_{21} }} + i}}{{{{\left( {\omega_{21} - \omega + n\Omega } \right)^{2} } \mathord{\left/ {\vphantom {{\left( {\omega_{21} - \omega + n\Omega } \right)^{2} } {\gamma_{21}^{2} }}} \right. \kern-\nulldelimiterspace} {\gamma_{21}^{2} }} + 1}}} .$$

Comparison of Eq. (22) with Eq. (15) shows that the nuclear response appearing at the combination frequencies is comparable to the nuclear response at the frequency of the incident field and hence should be taken into account in the case of relatively small vibration frequency $\Omega \sim \gamma_{21}$. The field appearing at the combination frequencies can distort the shape of the transmitted narrowband wave packet in such a way that the resulting propagation velocity and propagation delay may differ from the group velocity and group delay.

It should be noted that existing sources of 14.4-keV radiation considered below have the linewidths comparable to or larger than the linewidth of the ^57^Fe absorber (as shown in Fig. 2 by black dashed line). This means that in the most interesting case of a relatively low absorber vibration frequency promising the slowest group velocity, the group velocity and group delay model itself can be invalid for such relatively broadband wave packets. The real *propagation velocity* and *propagation delay* of the broadband wave packet in the vibrating absorber can differ from the group velocity and group delay of the narrowband wave packet. Nevertheless, as shown above, the steep resonant dispersion within the AIT window remains the basic physical mechanism of slowing down and delaying the incident wave packet once the major part of its spectrum is inside the AIT window.

## Group velocity in vibrating reference frame

**Figure 3:**
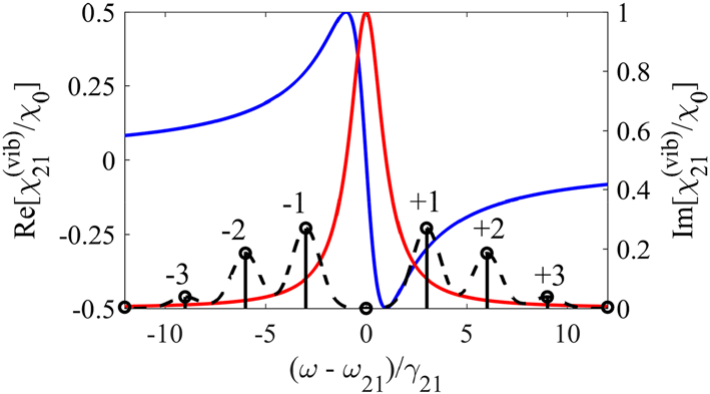
In the vibrating reference frame, real (blue curve, left axis) and imaginary (red curve, right axis) parts of the resonant susceptibility Eq. (23) of the motionless absorber as well as the power spectral density of the resonant, $\omega_{0} = \omega_{21}$, relatively broadened, $\Delta_{G} = \gamma_{21}$, incident wave packet with Gaussian spectral profile Eq. (36) (black dashed line) under ${\Omega \mathord{\left/ {\vphantom {\Omega {\gamma_{21} }}} \right. \kern-\nulldelimiterspace} {\gamma_{21} }} = 3$ and $p = p_{1} = 2.4$. The vertical black bars represent Fourier constituents with maximum amplitude at the frequencies $\omega_n=\omega_0+n\Omega$. In the case of a narrowband incident wave packet, the vertical black bars can be attributed to the discrete spectral components of the frequency-modulated field, having very narrow profiles. In the case under consideration, the plus- and minus-first broadened sidebands (black dashed line) are in the region of steep normal material dispersion.

As shown in^31,37,42,46,47^, the transition from the laboratory reference frame to the reference frame co-moving with the vibrating absorber ultimately gives the same results, but significantly simplifies calculations and allows for more transparent interpretation of the discussed effects. Indeed, in the vibrating reference frame, the absorber becomes motionless and has the Lorentzian spectral line of the resonant quantum transition with a HWHM $\gamma_{21}$ and a central frequency $\omega_{21}$ (Fig. 3),

23 $$\chi_{21}^{{\left( {vib} \right)}} \left( \omega \right) = \chi_{0} \frac{{{{\left( {\omega_{21} - \omega } \right)} \mathord{\left/ {\vphantom {{\left( {\omega_{21} - \omega } \right)} {\gamma_{21} }}} \right. \kern-\nulldelimiterspace} {\gamma_{21} }} + i}}{{{{\left( {\omega_{21} - \omega } \right)^{2} } \mathord{\left/ {\vphantom {{\left( {\omega_{21} - \omega } \right)^{2} } {\gamma_{21}^{2} }}} \right. \kern-\nulldelimiterspace} {\gamma_{21}^{2} }} + 1}}$$

The resonant susceptibility Eq. (23) immediately follows from Eq. (15) at $p_{1} = 0$ or from the well-known master equation for the nuclear coherence, $\rho_{21}$, of the two-level system, induced by the monochromatic field,

24 $$\frac{{\partial \rho_{21} }}{\partial t} + \left( {i\omega_{21} + \gamma_{21} } \right)\rho_{21} = \frac{i}{\hbar }n_{12} d_{21} \overline{E}(\omega ,z^{\prime})e^{{ - i\omega {\kern 1pt} t}} .$$

However, in the vibrating reference frame, the incident field Eq. (1) becomes frequency-modulated, $E(t,z^{\prime} = 0) = A(t,z^{\prime} = 0)\exp \left[ { - i\omega_{0} t + ik_{0} R\sin (\Omega t + \vartheta )} \right]$, which directly follows from substituting Eq. (4) into Eq. (1) and neglecting the harmonic modulation in the slowly varying amplitude of the quasi-monochromatic field (for more details, see^47^). Using Jacobi-Anger expansion, this frequency-modulated incident field can be represented as a set of partial wave packets with the carrier frequencies $\omega_{0} + n\Omega$, $n \in {\mathbb{Z}}$ and slowly varying amplitudes $A(t,z^{\prime} = 0)J_{ - n} (p)e^{ - in\vartheta }$,

25 $$E(t,z^{\prime} = 0) = A(t,z^{\prime} = 0)\sum\limits_{n = - \infty }^{\infty } {J_{ - n} \left( p \right)e^{{ - i(\omega_{0} + n\Omega )t}} e^{ - in\vartheta } } .$$

Their broadened spectral contours overlap (Fig. 3), forming a hilly-like resulting spectrum. Each Fourier constituent of the resulting spectrum is the superposition of contributions from all spectral contours,

26 $$\overline{E}(\omega ,z^{\prime}) = \frac{1}{2\pi }\int\limits_{ - \infty }^{\infty } {E\left( {t,z^{\prime}} \right)e^{i\omega t} dt} = \sum\limits_{n = - \infty }^{\infty } {J_{ - n} \left( p \right)e^{ - in\vartheta } S\left( {\omega - \omega_{n} ,z^{\prime}} \right)e^{{ik_{0} z^{\prime}}} } ,$$

where $\omega_{n} {\kern 1pt} = \omega_{0} + n\Omega$ is the carrier frequency of the *n*-th wave packet (the central frequency of the *n*-th spectral contour) and $S\left( {\omega ,z^{\prime}} \right) = \frac{1}{2\pi }\int\limits_{ - \infty }^{\infty } {A\left( {t,z^{\prime}} \right)e^{i\omega t} dt}$ is the spectral contour of the slowly varying amplitude of the parent wave packet.

In the case of a narrowband wave packet, namely $\Delta \ll \gamma_{21} ,\Omega$, the well-defined spectral components with narrow contours (the black vertical solid bars in Fig. 3) are separated by the frequency of the absorber vibration, $\Omega$. Their amplitudes and the corresponding spectral phases are determined by the Bessel function of the first kind, $J_{ - n} \left( p \right)$, where *n* is the number of a spectral component, and the initial phase of the absorber vibration, $\vartheta$. For the resonant incident field, $\omega_{0} = \omega_{21}$, its spectral components in the vibrating reference frame are arranged symmetrically with respect to $\omega_{21}$, whereas at $p = p_{1}$ the amplitude of the resonant spectral component is zero. If $\Omega > \gamma_{21}$, other components are in the range of relatively small resonant absorption (determined by ${\text{Im}} \left[ {\chi_{21}^{{\left( {vib} \right)}} \left( {\omega_{n} } \right)} \right]$ in Eq. (23)) and steep slope of the resonant normal dispersion (determined by ${\text{Re}} \left. {\left[ {{{d\chi_{21}^{{\left( {vib} \right)}} } \mathord{\left/ {\vphantom {{d\chi_{21}^{{\left( {vib} \right)}} } {d\omega }}} \right. \kern-\nulldelimiterspace} {d\omega }}} \right]} \right|_{{\omega = \omega_{n} }}$ in Eq. (23)) (Fig. 3). The latter leads to a decrease in the group velocities of the spectral components.

However, both the resonant absorption and the slope of the resonant dispersion of nuclei depend on the frequency and, therefore, are different for different spectral components. Both become smaller for $\pm (n + 1)$ spectral component compared to $\pm n$ spectral component (Fig. 3). As a result, according to Eq. (3), Eq. (20), Eq. (23), the *n*-th quasi-monochromatic spectral component experiences its own group delay due to the slope of the material dispersion,

27 $$\tau_{g}^{\left( n \right)} = \frac{{T_{a} }}{{2\gamma_{21} }} \cdot \frac{{\left( {{{n\Omega } \mathord{\left/ {\vphantom {{n\Omega } {\gamma_{21} }}} \right. \kern-\nulldelimiterspace} {\gamma_{21} }}} \right)^{2} - 1}}{{\left[ {\left( {{{n\Omega } \mathord{\left/ {\vphantom {{n\Omega } {\gamma_{21} }}} \right. \kern-\nulldelimiterspace} {\gamma_{21} }}} \right)^{2} + 1} \right]^{2} }},\quad n \ne 0.$$

Its own attenuation is determined by the absorber optical depth at frequency $\omega_{21} + n\Omega$:

28 $$T_{a}^{\left( n \right)} = {{4\pi \omega_{21} {\text{Im}} \left[ {\chi_{21}^{{\left( {vib} \right)}} \left( {\omega_{21} + n\Omega } \right)} \right]L} \mathord{\left/ {\vphantom {{4\pi \omega_{21} {\text{Im}} \left[ {\chi_{21}^{{\left( {vib} \right)}} \left( {\omega_{21} + n\Omega } \right)} \right]L} c}} \right. \kern-\nulldelimiterspace} c} = \frac{{T_{a} }}{{\left( {{{n\Omega } \mathord{\left/ {\vphantom {{n\Omega } {\gamma_{21} }}} \right. \kern-\nulldelimiterspace} {\gamma_{21} }}} \right)^{2} + 1}},\quad n \ne 0.$$

Comparison of Eq. (27) with Eq. (21) allows to assume that in the case of a narrowband wave packet and an optically thin absorber, the group delay, $\tau_{g}$, of the wave packet in the laboratory reference frame (Eq. (21)) is the weighted average of group delays of the spectral components, $\tau_{g}^{\left( n \right)}$, in the vibrating reference frame. As follows from Eqs. (28) and (19), the same correspondence is present between the effective resonant optical depth of the absorber in the laboratory reference frame, $T_{a}^{{\left( {eff} \right)}}$, and the absorber optical depths $T_{a}^{\left( n \right)}$, in the vibrating reference frame. This assumption will be verified in section "Slowing down Gaussian photons".

**Figure 4:**
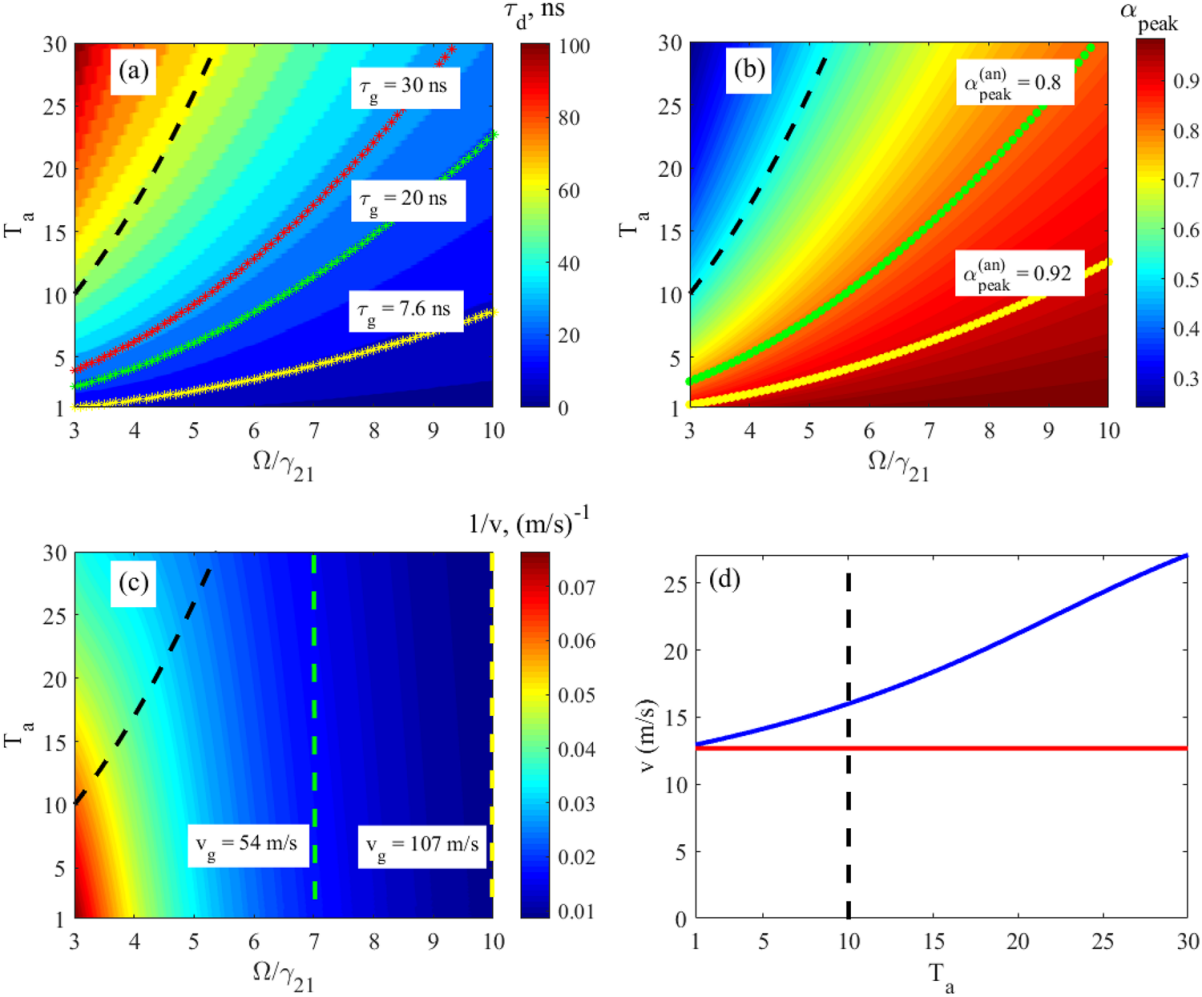
Characteristics of the relatively broadband Gaussian single-photon pulse (Eq. (36), where $\Delta_G=\gamma_{21}$) at the output from the vibrating absorber. **(a)** The moment (in nanoseconds) of formation of the pulse maximum, $\tau_{d}$, according to Eq. (34), **(b)** the normalized peak intensity of the transmitted pulse, $\alpha_{peak}$, according to Eq. (33), and **(c)** the reciprocal propagation velocity, ${1 \mathord{\left/ {\vphantom {1 v}} \right. \kern-\nulldelimiterspace} v}$, according to Eq. (35), as functions of the normalized absorber vibration frequency, ${\Omega \mathord{\left/ {\vphantom {\Omega {\gamma_{21} }}} \right. \kern-\nulldelimiterspace} {\gamma_{21} }}$, and the resonant optical depth, $T_{a}$ (see (17)) of the absorber plotted using numerical integration of Eq. (33). **(d)** The propagation velocity, blue line, of the same wave packet as a function of the resonant optical depth, $T_{a}$, at the fixed absorber vibration frequency, ${\Omega \mathord{\left/ {\vphantom {\Omega {\gamma_{21} }}} \right. \kern-\nulldelimiterspace} {\gamma_{21} }} = 3$, calculated using Eq. (35) and numerical integration of Eq. (33). Yellow, green and red stars in (**a**) mark the lines of constant group delay, $\tau_{g} = 7.6\;{\text{ns}}$, $20\;{\text{ns}}$, and $30\;{\text{ns}}$, respectively, estimated for a narrowband pulse, $\Delta_{G} \ll \gamma_{21}$, by Eqs. (40), (21). Yellow and green circles in (**b**) mark the lines of constant peak intensity of the transmitted pulse, $\alpha_{peak}^{(an)} = 0.92$ and $0.8$, respectively, estimated for a narrowband pulse, $\Delta_{G} \ll \gamma_{21}$, by Eqs. (43), (18). Green and yellow dashed lines in (**c**) correspond to the group velocities, $v_{g} = 54{\text{ m/s}}$, and $v_{g} = 107{\text{ m/s}}$, respectively, of the narrowband pulse, $\Delta_{G} \ll \gamma_{21}$ estimated by Eq. (19). On all panels, the black dashed lines mark the boundary $T_{a} = \left( {{\Omega \mathord{\left/ {\vphantom {\Omega {\gamma_{21} }}} \right. \kern-\nulldelimiterspace} {\gamma_{21} }}} \right)^{2} + 1$ (see Eq. (41)), approaching to which from the right and bottom in (**a**)–(**c**) and from the left in (**d**) invalidates the group velocity model. Other parameters of the absorber are presented after Eq. (19).

At the same time, in the case of an optically deep absorber, $T_{a} > 1$, and a low vibration frequency, $\Omega \sim \gamma_{21}$, this correspondence should vanish. Indeed, during propagation, the most intense and slowest plus- and minus-first narrowband spectral components (the vertical black bars in Fig. 3) are absorbed much stronger than other components. In the laboratory reference frame considered in section "Basic idea", this is equivalent to the generation of sidebands $\rho_{21}^{\left( q \right)}$ in the nuclear response (see Eq. (12)) and the appearance of a forward scattered field at the corresponding frequencies. As a result, the envelope of the wave packet may be reshaped. As follows from Fig. 3, the reduction of the slowest $\pm 1$ spectral components due to absorption will lead to a smaller pulse delay compared to the group delay, since the relative contribution of the $\pm 2, \pm 3,...$ spectral components, located on the less inclined nuclear dispersion, increases. The larger $T_{a}$, the smaller the $\pm 1$ spectral components and the smaller the pulse delay. This means that, in contrast to the group velocity independent of the absorber optical depth, the propagation velocity even for a narrowband wave packet must be higher than the group velocity estimated in the previous section and must increase with increasing the absorber optical depth (see also Fig. 4 below).

In the case of an incident wave packet with a broader linewidth typical for x-ray sources, different Fourier constituents of its spectrum (Fig. 3, black dashed line) experience different phase incursion and absorption depending on the spectral-temporal characteristics of the incident field, which corresponds to the group velocity dispersion and nonuniform absorption over the spectral contour. This difference increases at lower frequencies of the absorber vibration, which also leads to a change in the envelope of the transmitted wave packet and, can cause a change in the propagation velocity and delay. However, if a major part of the incident pulse spectrum in the vibrating reference frame is located in the range of normal nuclear dispersion (Fig. 3, blue line with a positive derivative), this dispersion ultimately leads to slowing down the pulse or, at least a significant part of it.

Thus, in the vibrating reference frame, the basic mechanism of photon slowing down is the same as in the laboratory reference frame, namely, it is a steep dispersion of the resonant nuclear transition, which can be accompanied by the above-mentioned effects. The correspondence between these reference frames is that the normal resonant dispersion at the frequency of the transparent nuclear transition for a single-frequency field in the laboratory reference frame is equivalent in the vibrating reference frame to the normal dispersion on the wings of the opaque nuclear transition for a set of spectral components of the transformed field, symmetrically detuned from the frequency of the nuclear transition to the region of low absorption. Different attenuation of the field spectral components due to different absorption in the vibrating reference frame is equivalent to the appearance of sidebands in the laboratory reference frame.

## Photon waveform in the vibrating absorber

All factors contributing to the photon propagation velocity are naturally taken into account in the temporal form of intensity (the photon waveform) at the exit from the absorber. In this section we calculate the intensity of spectrally broadened wave packet, Eq. (1), transmitted through the vibrating absorber. It is the intensity that is measured in the experiment and is used to determine the pulse delay in the medium. As is well-known and shown in^26,31,37,42,46,47,51^, for a weak field, in vibrating reference frame, the susceptibility of the absorber Eq. (23) at the frequency $\omega$ determines both the phase incursion and the amplitude of a monochromatic Fourier constituent at an arbitrary depth $z^{\prime}$ in the absorber according to the Beer–Lambert–Bouguer law,

29 $$\overline{E}(\omega ,z^{\prime}) = \overline{E}(\omega ,z^{\prime} = 0)e^{{ik_{0} z^{\prime}}} \exp \left[ {{{i2\pi \omega_{21} \chi_{21}^{{\left( {vib} \right)}} \left( \omega \right)z^{\prime}} \mathord{\left/ {\vphantom {{i2\pi \omega_{21} \chi_{21}^{{\left( {vib} \right)}} \left( \omega \right)z^{\prime}} c}} \right. \kern-\nulldelimiterspace} c}} \right],$$

which directly follows from the wave equation $c{{d\overline{E}(\omega ,z^{\prime})} \mathord{\left/ {\vphantom {{d\overline{E}(\omega ,z^{\prime})} {dz^{\prime}}}} \right. \kern-\nulldelimiterspace} {dz^{\prime}}} = i2\pi \omega_{21} \chi_{21}^{{\left( {vib} \right)}} \left( \omega \right)\overline{E}(\omega ,z^{\prime})$, where $n_{12} = {\text{Const}}$. For the case of frequency comb (see Eq. (26)), Eq. (29) turns to

30 $$\overline{E}(\omega ,z^{\prime}) = A_{0} \sum\limits_{n = - \infty }^{\infty } {J_{ - n} \left( p \right)e^{ - in\vartheta } S_{in} (\omega - \omega_{n} )} e^{{ik_{0} z^{\prime}}} \exp \left[ {{{i2\pi \omega_{21} \chi_{21}^{{\left( {vib} \right)}} \left( \omega \right)z^{\prime}} \mathord{\left/ {\vphantom {{i2\pi \omega_{21} \chi_{21}^{{\left( {vib} \right)}} \left( \omega \right)z^{\prime}} c}} \right. \kern-\nulldelimiterspace} c}} \right],$$

where $S_{in} (\omega ) \equiv {{S\left( {\omega ,z^{\prime} = 0} \right)} \mathord{\left/ {\vphantom {{S\left( {\omega ,z^{\prime} = 0} \right)} {A_{0} }}} \right. \kern-\nulldelimiterspace} {A_{0} }}$ is the spectral contour of the incident field in Eq. (26), normalized by its maximum value, $A_{0}$. Then the total field can be obtained by integrating Eq. (30) over $\omega$,

31 $$E(t,z^{\prime}) = A_{0} e^{{ik_{0} z^{\prime}}} \sum\limits_{n = - \infty }^{\infty } {J_{ - n} \left( p \right)e^{ - in\vartheta } \int\limits_{ - \infty }^{\infty } {S_{in} \left( {\omega - \omega_{n} } \right)\exp \left[ {{{i2\pi \omega_{21} \chi_{21}^{{\left( {vib} \right)}} \left( \omega \right)z^{\prime}} \mathord{\left/ {\vphantom {{i2\pi \omega_{21} \chi_{21}^{{\left( {vib} \right)}} \left( \omega \right)z^{\prime}} c}} \right. \kern-\nulldelimiterspace} c}} \right]e^{ - i\omega t} d\omega } } .$$

Substitution of Eq. (4) into Eq. (31) and neglecting the harmonic modulation in the slowly varying amplitude (for more details, see^47^) gives the field in the laboratory reference frame, which differs from Eq. (31) by $\exp \left[ { - ip\sin \left( {\Omega t + \vartheta } \right)} \right]$. Thus, the intensity of the field, $I(t,z^{\prime}) \propto \left| {E(t,z^{\prime})} \right|^{2}$, is the same in the laboratory and vibrating reference frames. At the exit from the absorber, the intensity $I_{out} (t) \equiv I(t,L)$ is

32 $$I_{out} (t) = \frac{{cA_{0}^{2} }}{8\pi }\left| {\sum\limits_{n = - \infty }^{\infty } {J_{ - n} \left( p \right)e^{ - in\vartheta } \int\limits_{ - \infty }^{\infty } {S_{in} \left( {\omega - \omega_{n} } \right)\exp \left[ { - \frac{{{{T_{a} } \mathord{\left/ {\vphantom {{T_{a} } 2}} \right. \kern-\nulldelimiterspace} 2}}}{{1 - {{i\left( {\omega - \omega_{21} } \right)} \mathord{\left/ {\vphantom {{i\left( {\omega - \omega_{21} } \right)} {\gamma_{21} }}} \right. \kern-\nulldelimiterspace} {\gamma_{21} }}}}} \right]e^{ - i\omega t} d\omega } } } \right|^{2} .$$

More detailed derivation of Eqs. (31) and (32) can be found in^31,37,46,47^.

As discussed above, the strongest slowing down in the AIT medium is expected for radiation having the smallest available spectral width. In the case of the 14.4-keV transition of ^57^Fe, the existing sources of narrowband resonant radiation suitable for AIT are ^57^Co radioactive Mössbauer sources^25–31^ and synchrotron Mössbauer sources^20–24^. Their minimum emission linewidth is close to the ^57^Fe absorption linewidth. In this case, both RMS and SMS can produce in a given direction only a sequence of single photons separated in time^20–31,37–39,42^. In the experiment, a single-photon wave packet Eq. (1) or Eq. (31) is formed as a result of measuring the number of the detected 14.4-keV photons per unit of time (i.e., count rate) as a function of time starting from a certain moment associated with the beginning of the single-photon wave packet. In the case of RMS ^57^Co, this moment is the detection of 122-keV photon that heralds the population of the emitting 14.4-keV state in the source. In the case of SMS, this moment is the emission time of SR pulse from the storage ring. The time dependence of the photon count rate is proportional to the time dependence of the photon detection probability, as well as to the intensity of the single-photon wave packet, Eq. (32). It is also called the photon waveform.

In the case of the vibrating ^57^Fe absorber, the initial vibration phase $\vartheta$ in Eq. (4) and the beginning of the single-photon wave packet can be matched^31,42,46,47^ or can be independent^37,46^. In the former case, the intensity of the single-photon wave packet transmitted through the AIT absorber is described by Eq. (32). The latter case is naturally realized with RMS due to stochastic emission of the radioactive source as well as with SMS if the absorber vibration frequency is not a multiple of the SR pulse repetition rate. In this case, the intensity in Eq. (32) should be averaged over the absorber initial vibration phase, $\vartheta$. Then if the carrier frequency of the 14.4-keV single-photon wave packet is in resonance with the ^57^Fe absorber transition, $\omega_{0}=\omega_{21}$, Eq. (32) can be written in the form

33 $${{\left\langle {I_{out} \left( t \right)} \right\rangle_{\vartheta } } \mathord{\left/ {\vphantom {{\left\langle {I_{out} \left( t \right)} \right\rangle_{\vartheta } } {I_{0} }}} \right. \kern-\nulldelimiterspace} {I_{0} }} = \sum\limits_{n = - \infty }^{\infty } {J_{n}^{2} \left( p \right)\left| {\int\limits_{ - \infty }^{\infty } {S_{in} \left( {\delta - n\Omega } \right)\exp \left\{ { - \frac{{{{T_{a} } \mathord{\left/ {\vphantom {{T_{a} } 2}} \right. \kern-\nulldelimiterspace} 2}}}{{1 - i{\delta \mathord{\left/ {\vphantom {\delta {\gamma_{21} }}} \right. \kern-\nulldelimiterspace} {\gamma_{21} }}}}} \right\}e^{ - i\delta t} d\delta } } \right|^{2} } ,$$

where $\delta = \omega - \omega_{21}$ is the detuning of the Fourier constituent, $\omega$, from the resonance, and $I_{0} = {{cA_{0}^{2} } \mathord{\left/ {\vphantom {{cA_{0}^{2} } {\left( {8\pi } \right)}}} \right. \kern-\nulldelimiterspace} {\left( {8\pi } \right)}}$. It should be noted that according to Eq. (25), the transmitted intensity, Eq. (33), constitutes a weighted average of the intensities of the single-photon wave packets $A(t,z^{\prime} = 0)e^{ - in\vartheta } e^{{ - i(\omega_{21} + n\Omega ){\kern 1pt} t}}$ after their independent transmission through the motionless absorber.

Below equation (33) is solved numerically, as well as analytically, under certain approximations discussed in section "Slowing down Gaussian photons". It determines the waveform of the transmitted photon, as well as the output peak intensity, $\alpha_{peak} \equiv {{\left\langle {I_{out} \left( {t_{\max }^{{\left( {out} \right)}} } \right)} \right\rangle_{{\vartheta_{0} }} } \mathord{\left/ {\vphantom {{\left\langle {I_{out} \left( {t_{\max }^{{\left( {out} \right)}} } \right)} \right\rangle_{{\vartheta_{0} }} } {I_{0} }}} \right. \kern-\nulldelimiterspace} {I_{0} }} = {\text{Max}}\left[ {{{\left\langle {I_{out} \left( t \right)} \right\rangle_{{\vartheta_{0} }} } \mathord{\left/ {\vphantom {{\left\langle {I_{out} \left( t \right)} \right\rangle_{{\vartheta_{0} }} } {I_{0} }}} \right. \kern-\nulldelimiterspace} {I_{0} }}} \right]$, and the moment when the output peak intensity occurs, $t_{\max }^{{\left( {out} \right)}}$.

The output peak intensity $\alpha_{peak}$ characterizes the absorption of the single-photon wave packet Eq. (33), transmitted through the absorber of length $L$. It should be noted that in experiments with a ^57^Fe absorber, the propagation of γ-ray or x-ray photons through the absorber is also accompanied by the non-resonant attenuation due to photoelectric effect. Therefore, the intensity Eq. (33) of the single-photon wave packet should be estimated as $\left\langle {I_{out}^{{\left( {{\text{exper}}} \right)}} \left( t \right)} \right\rangle_{\vartheta } = \left\langle {I_{out} \left( t \right)} \right\rangle_{\vartheta } \exp \left\{ { - \mu L} \right\}$, where *µ* is the linear photoelectric absorption coefficient. Below we consider a stainless-steel foil with *µ *≈ 5 × 10^4^ m^−1^^53^ and thickness below 1 µm. In this case, the difference between the experimentally measured intensity, $\left\langle {I_{out}^{{\left( {{\text{exper}}} \right)}} \left( t \right)} \right\rangle_{\vartheta }$, and the considered below intensity $\left\langle {I_{out} \left( t \right)} \right\rangle_{\vartheta }$ in Eq. (33), is less than 5%.

The moment $t_{\max }^{{\left( {out} \right)}}$ determines the corresponding propagation delay, $\tau_{d}$, of the pulse relative to the propagation time, $L/c$, in free space,

34 $$\tau_{d} \equiv t_{\max }^{{\left( {out} \right)}} - t_{\max }^{{\left( {in} \right)}} - {L \mathord{\left/ {\vphantom {L c}} \right. \kern-\nulldelimiterspace} c}\mathop \simeq \limits^{{\tau_{d} \gg {L \mathord{\left/ {\vphantom {L c}} \right. \kern-\nulldelimiterspace} c}}} t_{\max }^{{\left( {out} \right)}} - t_{\max }^{{\left( {in} \right)}} ,$$

where $t_{\max }^{{\left( {in} \right)}}$ is the time of the maximum intensity at the absorber entrance. The corresponding propagation velocity of the single-photon wave packet in the medium is defined as

35 $$v \equiv \frac{L}{{t_{\max }^{{\left( {out} \right)}} - t_{\max }^{{\left( {in} \right)}} }} = \frac{c}{{1 + {{c\tau_{d} } \mathord{\left/ {\vphantom {{c\tau_{d} } L}} \right. \kern-\nulldelimiterspace} L}}}\mathop \simeq \limits^{v \ll c} {L \mathord{\left/ {\vphantom {L {\tau_{d} }}} \right. \kern-\nulldelimiterspace} {\tau_{d} }}.$$

As discussed above and will be shown below, the propagation velocity and propagation delay of the single-photon wave packet emitted by the x-ray sources can differ from the group velocity and group delay.

## Slowing down Gaussian photons

Let’s first consider the model Gaussian 14.4-keV single-photon wave packet $E(t,z^{\prime} = 0) = A_{0} \exp \left( { - {{t^{2} \Delta_{G}^{2} } \mathord{\left/ {\vphantom {{t^{2} \Delta_{G}^{2} } 2}} \right. \kern-\nulldelimiterspace} 2}} \right)e^{{ - i\omega_{0} {\kern 1pt} t}}$, with spectral profile

36 $$S_{in} \left( \delta \right) = \frac{1}{{\Delta_{G} \sqrt {2\pi } }}\exp \left( { - \frac{{\delta^{2} }}{{2\Delta_{G}^{2} }}} \right).$$

The rapidly decreasing spectral wings of the Gaussian spectrum Eq. (36) make it possible to obtain an analytical solution of Eq. (33), ${{\left\langle {I_{out}^{{\left( {an} \right)}} \left( t \right)} \right\rangle_{\vartheta } } \mathord{\left/ {\vphantom {{\left\langle {I_{out}^{{\left( {an} \right)}} \left( t \right)} \right\rangle_{\vartheta } } {I_{0} }}} \right. \kern-\nulldelimiterspace} {I_{0} }}$, in the case of a relatively narrowband wave packet, $\Delta_{G} \ll \gamma_{21}$. Expansion of the absorber susceptibility in Eq. (33) in the Taylor series by ${{\left( {\delta - n\Omega } \right)} \mathord{\left/ {\vphantom {{\left( {\delta - n\Omega } \right)} {\gamma_{21} }}} \right. \kern-\nulldelimiterspace} {\gamma_{21} }}$ (i.e., in the vicinity of the central frequency $\omega_{n}$ of the *n*-th spectral component) to the linear term of the imaginary part and the constant term of the real part can be written as

37 $$\frac{{{{T_{a} } \mathord{\left/ {\vphantom {{T_{a} } 2}} \right. \kern-\nulldelimiterspace} 2}}}{{1 - i{\delta \mathord{\left/ {\vphantom {\delta {\gamma_{21} }}} \right. \kern-\nulldelimiterspace} {\gamma_{21} }}}} \simeq \frac{{T_{a}^{\left( n \right)} }}{2} + i\frac{n\Omega }{{2\gamma_{21} }}T_{a}^{\left( n \right)} - i\left( {\delta - n\Omega } \right)\tau_{g}^{\left( n \right)} .$$

This is equivalent to assumption of the linear dispersion of the absorber on the scale $\Delta_{G}$ and uniform (as a whole) absorption of the contour $S_{in} \left( {\delta - n\Omega } \right)$ of the *n*-th spectral component. In other words, in this approximation, the corresponding partial wave packet $A_{n} (t,z^{\prime})e^{{ - i(\omega_{n} {\kern 1pt} t - k_{0} z^{\prime})}}$ of the sum given by Eq. (25) propagates with group velocity $v_{g}^{(n)} \simeq {L \mathord{\left/ {\vphantom {L {\tau_{g}^{\left( n \right)} }}} \right. \kern-\nulldelimiterspace} {\tau_{g}^{\left( n \right)} }}$ (see also Eq. (27)) determined by the slope of the nuclear dispersion at frequency $\omega_n$, and is absorbed as a whole with the absorption coefficient ${{T_{a}^{(n)} } \mathord{\left/ {\vphantom {{T_{a}^{(n)} } 2}} \right. \kern-\nulldelimiterspace} 2}$ (see also Eq. (28)), as a narrowband wave packet. In Fig. 3 this approximation corresponds to vertical black bars. In this case, one can write the intensity Eq. (33) of the transmitted single-photon wave packet Eq. (36) in the form,

38 $${{\left\langle {I_{out}^{{\left( {an} \right)}} \left( t \right)} \right\rangle_{\vartheta } } \mathord{\left/ {\vphantom {{\left\langle {I_{out}^{{\left( {an} \right)}} \left( t \right)} \right\rangle_{\vartheta } } {I_{0} }}} \right. \kern-\nulldelimiterspace} {I_{0} }} = \sum\limits_{n = - \infty }^{\infty } {J_{n}^{2} \left( {p_{1} } \right)\exp \left( { - T_{a}^{\left( n \right)} } \right)\exp \left[ { - \Delta_{G}^{2} \left( {t - \tau_{g}^{\left( n \right)} } \right)^{2} } \right]} .$$

The maximum of function Eq. (38) corresponds to the moment of the pulse peak formation, $t_{\max }^{{\left( {out} \right)}}$, which, according to Eq. (34), determines the photon propagation delay $t_{\max }^{{\left( {out} \right)}} \simeq \tau_{d}$ (since $t_{\max }^{{\left( {in} \right)}} = 0$). Equating the time derivative of Eq. (38) to zero gives the relation

39 $$\tau_{d} \sum\limits_{n = - \infty }^{\infty } {J_{n}^{2} \left( {p_{1} } \right)e^{{ - T_{a}^{\left( n \right)} }} e^{{ - \Delta_{G}^{2} \left( {\tau_{d} - \tau_{g}^{\left( n \right)} } \right)^{2} }} } = \sum\limits_{n = - \infty }^{\infty } {J_{n}^{2} \left( {p_{1} } \right)\tau_{g}^{\left( n \right)} e^{{ - T_{a}^{\left( n \right)} }} e^{{ - \Delta_{G}^{2} \left( {\tau_{d} - \tau_{g}^{\left( n \right)} } \right)^{2} }} } .$$

Solution of Eq. (39) coincides with the group delay Eq. (21), and verifies our assumption in section "Group velocity in vibrating reference frame" about the weighted average,

40 $$\tau_{d} \simeq \sum\limits_{n = - \infty }^{\infty } {J_{n}^{2} \left( {p_{1} } \right)\tau_{g}^{\left( n \right)} } = \tau_{g} ,$$

provided two conditions are fulfilled: (1) the absorber vibration frequency is high enough and/or the absorber optical depth is small enough to provide sufficiently low absorption for the plus- and minus-first frequency components, $T_{a}^{\left( 1 \right)} \ll 1$ or

41 $$\frac{{T_{a} }}{{\left( {{\Omega \mathord{\left/ {\vphantom {\Omega {\gamma_{21} }}} \right. \kern-\nulldelimiterspace} {\gamma_{21} }}} \right)^{2} + 1}} \ll 1,$$

and (ii) the group delay of the plus- and minus-first spectral components, which primarily determines the delay of the single-photon wave packet, is less than the duration of the incident wave packet, $\tau_{g}^{(1)} \Delta_{G} \ll 1$, or

42 $$\frac{{\Delta_{G} }}{{\gamma_{21} }} \ll \frac{2}{{T_{a} }}\;\frac{{\left[ {\left( {{\Omega \mathord{\left/ {\vphantom {\Omega {\gamma_{21} }}} \right. \kern-\nulldelimiterspace} {\gamma_{21} }}} \right)^{2} + 1} \right]^{2} }}{{\left| {\left( {{\Omega \mathord{\left/ {\vphantom {\Omega {\gamma_{21} }}} \right. \kern-\nulldelimiterspace} {\gamma_{21} }}} \right)^{2} - 1} \right|}}\mathop \simeq \limits^{{{\Omega \mathord{\left/ {\vphantom {\Omega {\gamma_{21} }}} \right. \kern-\nulldelimiterspace} {\gamma_{21} }} \gg 1}} \frac{2}{{T_{a} }}\left( {\frac{\Omega }{{\gamma_{21} }}} \right)^{2} .$$

The condition given by Eq. (41) corresponds to the condition $T_{a}^{{\left( {eff} \right)}} \ll 1$ and allows neglecting the absorption of spectral components. The condition given by Eq. (42) limits the spectral width of the incident wave packet taking into account the vibration frequency and optical depth of the absorber. At this condition, the group delays of the strongest plus- and minus-first partial wave packets in sum of Eq. (25), $A_{0} \exp \left( { - {{t^{2} \Delta_{G}^{2} } \mathord{\left/ {\vphantom {{t^{2} \Delta_{G}^{2} } 2}} \right. \kern-\nulldelimiterspace} 2}} \right)J_{ \mp 1} \left( {p_{1} } \right)e^{ \mp i\vartheta } e^{{ - i\left( {\omega_{0} \pm \Omega } \right){\kern 1pt} t}}$, after passing through the absorber do not exceed their durations. Thus, according to Eqs. (27) and (28), conditions Eqs. (41) and (42) are sufficient for the validity of Eq. (40) and the assumption in section "Group velocity in vibrating reference frame", since the plus- and minus-first spectral components are the strongest and closest to resonance.

The relations given by Eq. (41), Eq. (42) limit the range of the parameter values at which the propagation of the Gaussian wave packet through the AIT-medium is well characterized by the group velocity Eq. (19), $v \simeq v_{g}$, and group delay Eq. (21), $\tau_{d} \simeq \tau_{g}$, and may be described within the model of the AIT spectral window in the laboratory reference frame (see section "Basic idea"). In this parameter area, the peak intensity of the transmitted single-photon wave packet, following from Eqs. (38) and (40), is

43 $$\alpha_{peak}^{(an)} \equiv \left[ {{{\left\langle {I_{out}^{{\left( {an} \right)}} \left( t \right)} \right\rangle_{{\vartheta_{0} }} } \mathord{\left/ {\vphantom {{\left\langle {I_{out}^{{\left( {an} \right)}} \left( t \right)} \right\rangle_{{\vartheta_{0} }} } {I_{0} }}} \right. \kern-\nulldelimiterspace} {I_{0} }}} \right]_{\max } \simeq 1 - \sum\limits_{n = - \infty }^{\infty } {J_{n}^{2} \left( {p_{1} } \right)T_{a}^{\left( n \right)} } = 1 - T_{a}^{{\left( {eff} \right)}} .$$

Equation (43) constitutes the Beer–Lambert–Bouguer law for a resonant absorber that has become transparent due to AIT.

Let’s now consider the propagation delay, $\tau_{d}$, propagation velocity, $v$, and output peak intensity, $\alpha_{peak}$, of the relatively broadband, $\Delta_{G} = \gamma_{21}$, Gaussian 14.4-keV wave packet, Eq. (36), in the vibrating ^57^Fe absorber, obtained by numerical integration of Eq. (33), for various absorber optical depths and vibration frequencies (Fig. 4). The absorber is the discussed above stainless-steel foil Fe_70_Cr_19_Ni_11_ 95% enriched with ^57^Fe nuclei having the natural linewidth, ${{\gamma_{21} } \mathord{\left/ {\vphantom {{\gamma_{21} } {\left( {2\pi } \right)}}} \right. \kern-\nulldelimiterspace} {\left( {2\pi } \right)}} = 0.56\;{\text{MHz}}$, of the 14.4-keV resonant transition. For comparison, in Fig. 4, we also plotted analytical estimates for the group delay according to Eq. (21), group velocity according to Eq. (19), and peak intensity according to Eq. (43) of the narrowband Gaussian pulse.

As indicated by the relations in Eqs. (41) and (42), these analytical estimates can be considered only in the region of sufficiently large absorber vibration frequency and small optical depth i.e., far lower and to the right from the black dashed lines in Fig. 4a–c and to the left in Fig. 4d. As can be seen, in these regions, the analytical estimates for the narrowband wave packet are in rather good agreement with the numerical estimates for the broadband wave packet, $\Delta_{G} = \gamma_{21}$, (the starred, dotted and dashed colored lines are mostly within certain colors). Therefore, the propagation delay and propagation velocity of a broadband single-photon wave packet with a spectrum sufficiently narrower than the AIT window in an absorber with a sufficiently small optical depth indeed tend to group delay (Eq. (21)) and group velocity (Eq. (19)) due to the steep dispersion of the absorber, as discussed in section "Basic idea".

As can be seen in Fig. 4a,c at any fixed optical depth of the absorber, a decrease in the vibration frequency leads to an increase in the propagation delay and, accordingly, to a decrease in the propagation velocity of the broadband Gaussian pulse due to a steeper nuclear dispersion. However, at the same time, the peak pulse intensity decreases (Fig. 4b) due to higher absorption. At a very low vibration frequency, a significant part of the field spectrum can fall into the region of anomalous nuclear dispersion and high absorption (see Fig. 3 in the vibrating reference frame and Fig. 2 in the laboratory reference frame), which leads to a large distortion of the photon waveform and a large decrease in the output intensity. Hence, the minimum allowable vibration frequency, $\Omega_{\min }$, of the absorber is limited jointly by the linewidth of the nuclear resonance and the spectral width of the wave packet in such a way that most part of the plus- and minus-first spectral sidebands are located in the region of the normal material dispersion (Fig. 3). This condition is met if

44 $${{\Omega_{\min } } \mathord{\left/ {\vphantom {{\Omega_{\min } } {(\Delta_{G} + \gamma_{21} )}}} \right. \kern-\nulldelimiterspace} {(\Delta_{G} + \gamma_{21} )}} = 1.5.$$

Thus, Eq. (44) determines the absorber vibration frequency for achieving the greatest slowing down of photon via steep nuclear dispersion. In the case $\Delta_{G} = \gamma_{21}$, Eq. (44) is read as ${{\Omega_{\min } } \mathord{\left/ {\vphantom {{\Omega_{\min } } {\gamma_{21} }}} \right. \kern-\nulldelimiterspace} {\gamma_{21} }} = 3$.

**Figure 5:**
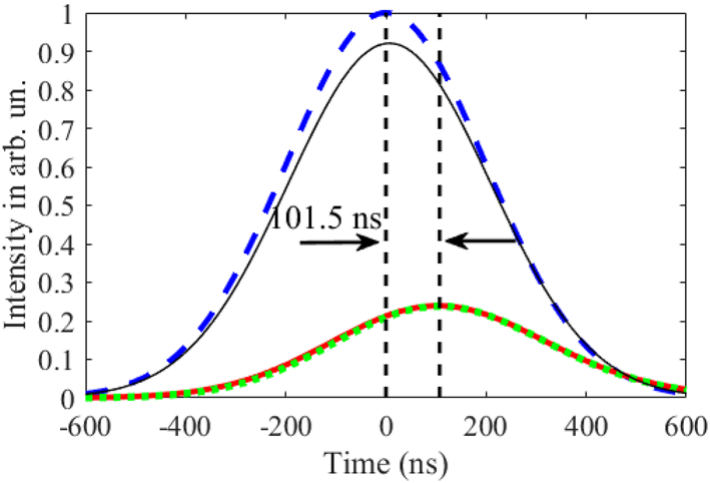
Time dependence of the intensity of the 14.4-keV Gaussian single-photon wave packet Eq. (36) at the entrance (blue dashed line) and exit from the absorber vibrating at the frequency ${\Omega \mathord{\left/ {\vphantom {\Omega {\gamma_{21} }}} \right. \kern-\nulldelimiterspace} {\gamma_{21} }} = 3$ with amplitude $R_{1} = 0.38\lambda$, Eq. (14), corresponding to $p_{1} = 2.4$, Eq. (13), as well as for ${{\Delta_{G} } \mathord{\left/ {\vphantom {{\Delta_{G} } {\left( {2\pi } \right)}}} \right. \kern-\nulldelimiterspace} {\left( {2\pi } \right)}} = {{\gamma_{21} } \mathord{\left/ {\vphantom {{\gamma_{21} } {\left( {2\pi } \right)}}} \right. \kern-\nulldelimiterspace} {\left( {2\pi } \right)}} = 0.56\;{\text{MHz}}$. The black solid thin line corresponds to $T_{a} = 1$, whereas the red solid and green dotted lines correspond to $T_{a} = 30$. The black and red solid lines are calculated by the numerical integration of Eq. (33), whereas the green dotted line is calculated using Eq. (38) without taking into account the contributions of both the group velocity dispersion and the frequency-dependence of the absorption within each spectral component.

At this vibration frequency, according to Eqs. (41), (42), the model of the group velocity independent of $T_{a}$ is valid only for optically thin absorber, i.e., at $T_{a} < 1$. In an optically thicker absorber, photons are characterized by the propagation velocity, which we obtain by numerical integration of Eq. (33). As expected, the propagation velocity is higher than the group velocity (Fig. 4d), since $\pm n$-th spectral components in the vibrating reference frame are attenuated stronger than $\pm (n + 1)$-th components (see Fig. 3 and the last three paragraphs in section "Group velocity in vibrating reference frame"). At larger $T_{a}$, this difference in the absorption of spectral components becomes greater leading to increase in the pulse propagation velocity (Fig. 4d). In such a way, the lowest propagation velocity can be achieved at the smallest $T_{a}$, tending to the group velocity at $T_{a} < 1$. In this case, the intensity and shape of the pulse are preserved. For example, at $T_{a} = 1$ the propagation velocity is $v \approx 12.5{\text{ m/s}}$ (Fig. 4c,d), which is close to the group velocity estimated in section "Basic idea", whereas the peak pulse intensity is $\alpha_{peak} \approx 0.92$ (Fig. 4b yellow circles and Fig. 5 black solid line).

It should be noted that the small optical depth of the absorber corresponds to a small physical thickness (according to Eq. (17), $T_{a} = \sigma_{0} f_{a} Nn_{12} L$) and hence to a small delay of the pulse. For example, the thickness of our stainless-steel foil Fe_70_Cr_19_Ni_11_, 95% enriched with ^57^Fe and having $T_{a} = 1$, is as small as $L \approx 95{\text{ nm}}$. The corresponding propagation delay, Eq. (35), of the Gaussian pulse, obtained from the numerical integration of Eq. (33), is only $\tau_{d} \approx 7.6{\text{ ns}}$ (Fig. 4a, yellow stars and Fig. 5 black solid line). A much larger delay, namely $\tau_{d} \approx 101.5{\text{ ns}}$ (Fig. 4a, upper left corner), accompanied by a relatively modest attenuation, $\alpha_{peak} \approx 0.24$ (Fig. 4b, upper left corner and Fig. 5), can be achieved with the absorber optical depth $T_{a} = 30$ corresponding to $L \approx 2.85 \, \mu {\text{m}}$. This pulse delay corresponds to the propagation velocity $v \approx 28{\text{ m/s}}$. The waveform of the delayed Gaussian photon is shown in Fig. 5. It almost repeats the waveform of the incident photon. At the same time, stronger absorption of the spectral components closer to the resonance causes not only an increase in the propagation velocity, but also some lengthening of the pulse.

The delayed pulse in Fig. 5 is obtained both by numerical integration of Eq. (33) (red solid line) and by calculation using Eq. (38) (green dotted line). The latter assumes that, despite $\Delta_{G} = \gamma_{21}$, in the vibrating reference frame there is no group velocity dispersion and no change in the field absorption on the scale of the *n*-th ($n = \pm 1, \pm 2,...$) spectral component (see also Fig. 3). The almost complete coincidence of the curves shows that the basic mechanism for slowing down and delaying this Gaussian pulse in the vibrating absorber is the steep normal nuclear dispersion on the wings of the spectral contour of the nuclear transition, accompanied by nuclear absorption at a high optical depth of the absorber in the co-moving reference frame.

In conclusion of this section, the slowest propagation velocity on the order of several tens of meters per second and the largest delay of about one hundred nanoseconds of the 14.4-keV Gaussian pulse with a relatively modest attenuation of intensity and preservation of its shape can be achieved at the lowest ^57^Fe absorber vibration frequency (limited by the sum of the nuclear transition linewidth and field linewidth), providing the steepest nuclear dispersion. The accompanying attenuation of the field leads to a higher propagation velocity of the pulse compared to the group velocity and slight lengthening. They increase with an increase in the absorber optical depth. Along with this, the pulse delay also increases.

The Gaussian shape of the pulse provides the clearest physical picture of photon slowing down and delay due to AIT. However, the Gaussian pulses with photon energy of 14.4 keV suitable for slowing down and delay in the vibrating ^57^Fe absorber are not available yet. The available radioactive or synchrotron sources of 14.4-keV radiation produce photons with Lorentz or Lorentz-like spectral profiles. Slowing down of such photons in the vibrating resonant ^57^Fe absorber under AIT conditions is studied in the next two sections.

## Slowing down Lorentzian photons

In this section, we consider the propagation of 14.4-keV photons with the Lorentz spectrum,

45 $$S_{in} \left( \delta \right) = \frac{1}{2\pi }\frac{1}{{\gamma_{S} - i\delta }},$$

through the vibrating single-line ^57^Fe absorber under the above conditions of AIT Eqs. (13), (14). Such photons are typically emitted by RMS ^57^Co as well as by SMS at certain conditions^21,23^ The RMS ^57^Co linewidth is usually comparable to the ^57^Fe absorber resonance linewidth. Thus, as a limiting case, we assume the natural linewidths of both the source and absorber, ${{\gamma_{S} } \mathord{\left/ {\vphantom {{\gamma_{S} } {(2\pi )}}} \right. \kern-\nulldelimiterspace} {(2\pi )}} = {{\gamma_{21} } \mathord{\left/ {\vphantom {{\gamma_{21} } {(2\pi )}}} \right. \kern-\nulldelimiterspace} {(2\pi )}} \approx 0.56{\text{ MHz}}$. The spectrum Eq. (45) corresponds to the exponentially decaying incident single-photon wave packet Eq. (1) with a stepwise front,

46 $$A(t,0) = \theta \left( t \right)\exp \left( { - \gamma_{21} t} \right),$$

where $\theta \left( t \right)$ is the unit step function. The long spectral wings corresponding to the stepwise front edge of the single-photon wave packet Eqs. (45), (46), propagate through the absorber almost without resonant interaction. In the motionless absorber, this corresponds to formation of a Sommerfeld-Brillouin precursor, described by equation ${{\left\langle {I_{out} \left( t \right)} \right\rangle_{\vartheta } } \mathord{\left/ {\vphantom {{\left\langle {I_{out} \left( t \right)} \right\rangle_{\vartheta } } {I_{0} }}} \right. \kern-\nulldelimiterspace} {I_{0} }} = \theta \left( t \right)e^{{ - \Gamma_{21} t}} J_{0}^{2} \left( {\sqrt {\Gamma_{21} T_{a} t} } \right)$ (where $\Gamma_{21} = 2\gamma_{21}$)^25,51^, in the output photon waveform, which follows from Eq. (33). It has the stepwise front edge and its maximum is not delayed. Its half-height duration can either be much shorter than the duration of the incident pulse, ${{\tau_{SB} \approx 1} \mathord{\left/ {\vphantom {{\tau_{SB} \approx 1} {\left( {\Gamma_{21} T_{a} } \right)}}} \right. \kern-\nulldelimiterspace} {\left( {\Gamma_{21} T_{a} } \right)}}$ (called the speedup effect^51^), if $T_{a} \gg 1$, or approach the incident pulse duration, ${{\tau_{SB} \approx 1} \mathord{\left/ {\vphantom {{\tau_{SB} \approx 1} {\Gamma_{21} }}} \right. \kern-\nulldelimiterspace} {\Gamma_{21} }}$, if $T_{a} \ll 1$. Thus, the polarization response of the medium to the Lorentzian pulse is delayed, being formed during the time $\tau_{SB}$. As a result, in contrast to the Gaussian pulse delayed in the AIT-absorber as a whole, the Lorentzian single-photon wave packet should be split into two parts: the Sommerfeld-Brillouin precursor passed without delay, and the next delayed part due to the field-nuclei interaction (see also Fig. 7). The total delay of the delayed part of the pulse is summed up from the delayed nuclear response and the delay due to the real part of the nuclear response corresponding to dispersion of the nuclear transition. Taking this into account, we estimate the propagation delay, $\tau_{d}$, Eq. (34), and propagation velocity, $v$, Eq. (35), of the delayed part of the Lorentzian wave packet by numerical calculation of the intensity, Eq. (33), where $\tau_{d} = t_{\max }^{{\left( {out} \right)}}$ is the moment of time corresponding to the first maximum in the transmitted photon waveform, Eq. (33), following the initial moment corresponding to the undelayed peak of the Sommerfeld-Brillouin precursor, $t_{\max }^{{\left( {in} \right)}} = 0$.

**Figure 6:**
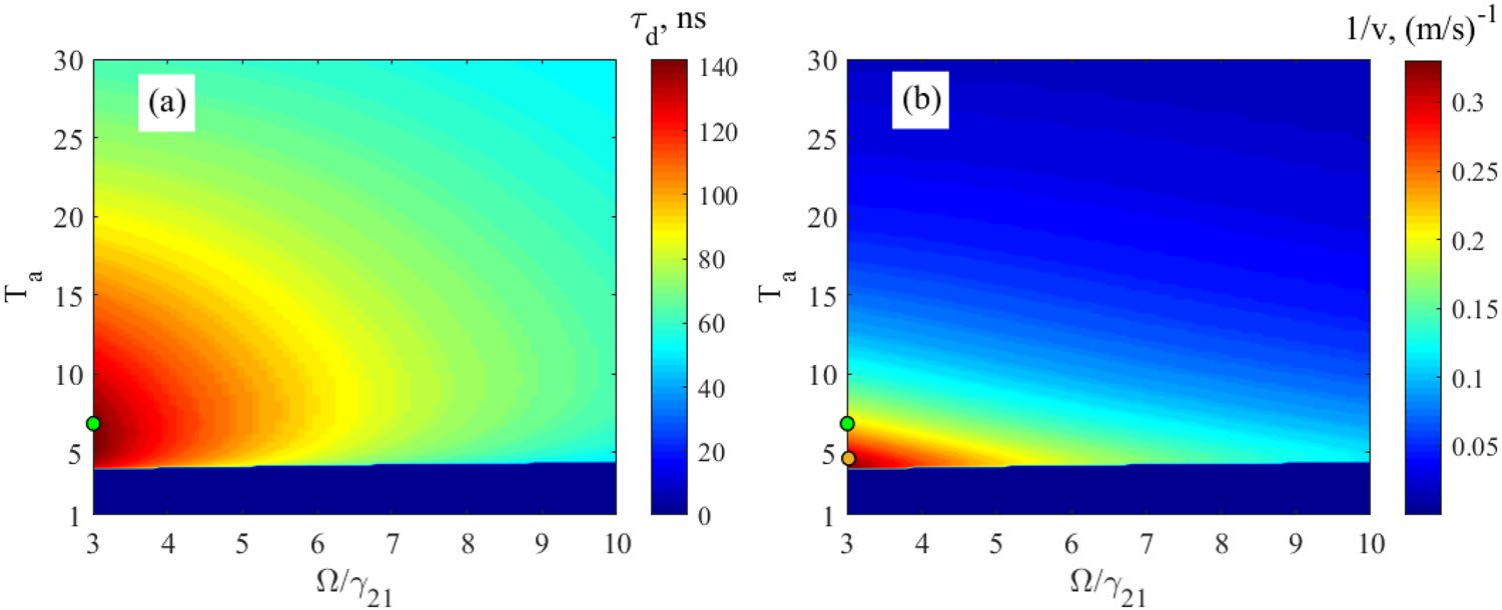
(**a**) The moment (in nanoseconds) of formation of the delayed pulse maximum, $\tau_{d}$, as a function of the absorber normalized vibration frequency, ${\Omega \mathord{\left/ {\vphantom {\Omega {\gamma_{21} }}} \right. \kern-\nulldelimiterspace} {\gamma_{21} }}$, and the resonant optical depth, $T_{a}$ (see Eq. (17)), plotted using numerical integration of Eq. (33) taking into account Eq. (45). The green circle marks the longest photon delay of 144 ns. (**b**) Dependence of the reciprocal propagation velocity, ${1 \mathord{\left/ {\vphantom {1 v}} \right. \kern-\nulldelimiterspace} v}$ in (m/s)^−1^, on the absorber vibration frequency, ${\Omega \mathord{\left/ {\vphantom {\Omega {\gamma_{21} }}} \right. \kern-\nulldelimiterspace} {\gamma_{21} }}$, and the optical depth, $T_{a}$, calculated according to Eq. (35) and panel (**a**). As above, the absorber is a stainless-steel foil Fe_70_Cr_19_Ni_11_, 95% enriched with ^57^Fe nuclide, vibrating with amplitude $R_{1}$ in Eq. (14) corresponding to $p = p_{1}$ in Eq. (13). The green circle marks the propagation velocity of photons having the longest photon delay, which corresponds to the red waveform in Fig. 7 and equals 4 m/s. The orange circle marks the minimum propagation velocity of Lorentzian photons equal to 3 m/s. In the dark blue regions, both a dip and a local maximum after the Sommerfeld-Brillouin precursor are absent (the derivative changes value but does not change sign), therefore zero value is set in the numerical simulation. Above the boundaries of the blue regions, both a dip and a local maximum after the Sommerfeld-Brillouin precursor are numerically identified.

**Figure 7:**
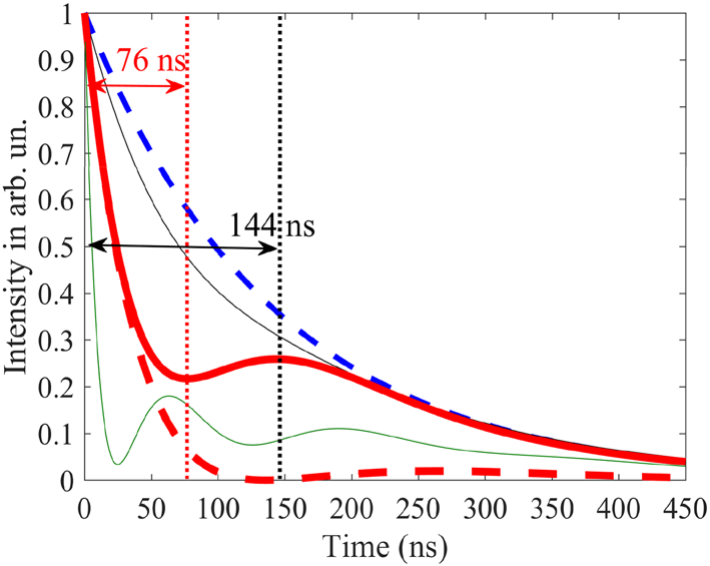
Waveforms of the of 14.4-keV Lorentzian single-photon wave packet. The blue dashed line corresponds to the incident photon waveform emitted by ^57^Co RMS with ${{\gamma_{S} } \mathord{\left/ {\vphantom {{\gamma_{S} } {\left( {2\pi } \right)}}} \right. \kern-\nulldelimiterspace} {\left( {2\pi } \right)}} = 0.56\;{\text{MHz}}$. Other lines are the waveforms at the exit from the ^57^Fe absorber with $\gamma_{21} = \gamma_{S}$. The red dashed line corresponds to the motionless absorber with $T_{a} = 6$ in comparison with the red solid line for the same absorber vibrating at the frequency ${\Omega \mathord{\left/ {\vphantom {\Omega {\gamma_{21} }}} \right. \kern-\nulldelimiterspace} {\gamma_{21} }} = 3$ with amplitude $R_{1}$ ($p_{1} = 2.4$, see Eqs. (13), (14)). The red solid line has the Sommerfeld-Brillouin precursor with duration ${{\tau_{SB} \approx 1} \mathord{\left/ {\vphantom {{\tau_{SB} \approx 1} {\left( {2\gamma_{21} T_{a} } \right)}}} \right. \kern-\nulldelimiterspace} {\left( {2\gamma_{21} T_{a} } \right)}} \approx 24\;{\text{ns}}$ at half-height, transmitted without delay, and the delayed part of the single-photon wave packet. The green line demonstrates the dynamical beats in the waveform of the photon passed through the same vibrating absorber with $T_{a} = 30$, where the concept of the propagation delay and velocity becomes inapplicable. The black solid thin line is plotted for the same vibrating absorber with $T_{a} = 1$. All curves are plotted using numerical integration of Eq. (33).

In Fig. 6 we show the dependences of the delay and reciprocal propagation velocity on the absorber optical depth, $T_{a}$, and its normalized vibration frequency, ${\Omega \mathord{\left/ {\vphantom {\Omega {\gamma_{21} }}} \right. \kern-\nulldelimiterspace} {\gamma_{21} }}$. In contrast to the case of the Gaussian photon, not two but three regions can be distinguished in each panel of Fig. 6. The dark blue region of relatively large absorber vibration frequency and small optical depth, Eq. (41), corresponds to high AIT, which was realized in^37^. A transmitted photon waveform, typical for high AIT, is shown in Fig. 7 by the thin black solid line. It is close to the exponential shape of the incident photon (blue dashed line in Fig. 7), since the half-height duration of the Sommerfeld-Brillouin precursor in this case is close to the duration of the incident photon, 144 ns, and the subsequent delay due to nuclear dispersion is only about 8 ns, which is indistinguishable at the scale of the picture. This delay is approximately the same as the propagation delay of the Gaussian photon at these parameter values (see Fig. 5, solid black line) and is close to the group delay. In the numerical simulation used, this propagation delay is not identified, since in this case there is neither a dip nor a local maximum in the transmitted photon waveform after the front edge (the derivative changes value but does not change sign). This is also the reason for the contrast boundaries of the dark blue regions in Fig. 6, above which the local maximum begins to be identified.

**Figure 8:**
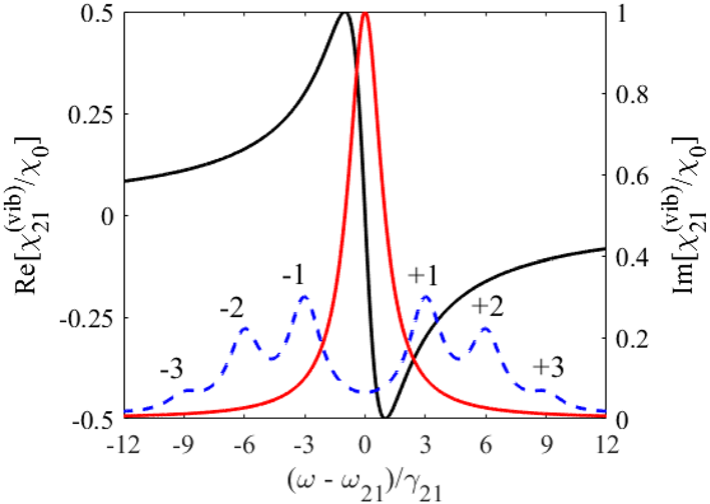
In the vibrating reference frame, real (black curve, left axis) and imaginary (red curve, right axis) parts of the resonant susceptibility Eq. (23) of the motionless absorber as well as the spectral density of the resonant, $\omega_0=\omega_{21}$, spectrally broadened, $\gamma_{S} = \gamma_{21}$, incident wave packet with Lorentz spectral profile, Eq. (45), (blue dashed line) under ${\Omega \mathord{\left/ {\vphantom {\Omega {\gamma_{21} }}} \right. \kern-\nulldelimiterspace} {\gamma_{21} }} = 3$ and $p=p_1=2.4$.

In the regions near the upper left corners in Fig. 6a,b corresponding to large optical depth and low absorber vibration frequency, the vibrating absorber remains optically thick, $T_{a}^{{\left( {eff} \right)}} > 1$. For example, the green line in Fig. 7 corresponds to $T_{a}^{{\left( {eff} \right)}} \approx 2$. In this case, a large attenuation of a relatively strong resonant part of the field (due to overlapped long wings of the Lorentz spectral contours) in the vibrating reference frame (Fig. 8), leads to the so-called dynamical beats in the waveform of the output photon waveform^25^ (Fig. 7, green line). As a result, the distortions in the incident photon spectrum and waveform are so large that the concept of the pulse delay and pulse propagation velocity become inapplicable in this region.

As shown in Fig. 6, the maximum propagation delay and the minimum propagation velocity of the Lorentzian pulse occur at the minimum absorber vibration frequency, limited by relation (44), ${\Omega \mathord{\left/ {\vphantom {\Omega {\gamma_{21} }}} \right. \kern-\nulldelimiterspace} {\gamma_{21} }} = 3$, as in the case of the Gaussian photon. However, unlike the Gaussian photon, in the case of the Lorentzian photon, there is also an optimal optical depth, $T_{a} \approx 6$, at which the propagation delay achieves the maximum value (Fig. 6a, green circle), whereas the propagation delay of the Gaussian photon constantly increases with increasing optical depth (see Fig. 4a). The maximally delayed Lorentzian single-photon pulse at optimal values of $\Omega$ and $T_{a}$ is shown in Fig. 7 by the solid red line. It consists of two maxima. The first maximum at $t = 0$ corresponds to the peak of the undelayed Sommerfeld–Brillouin precursor, and the second maximum corresponds to the delayed fraction of the output wave packet caused by steep nuclear dispersion inside the AIT spectral window in the formed nuclear response (see also Fig. 2, where the blue and red lines are the nuclear response, and the dashed black line is the incident field spectrum). The latter can be seen from a comparison of the red solid line in Fig. 7 with the dashed red line corresponding to the same photon passed through the same absorber at rest. In the motionless absorber, the incident photon is completely absorbed during 136 ns due to the rapidly developing collective response of nuclei (speed-up effect^51^), which is characterized by strong absorption of the Lorentz-shape field resonant to the nuclear transition. The collective response of the oscillating nuclei to the stepwise front (long spectral wings) of the pulse is the same as for the motionless absorber, namely strong and fast, which corresponds to the same shape and duration of the Sommerfeld–Brillouin precursor and strong attenuation of the field. However, as soon as the nuclear response becomes sufficiently developed (in about 60 ns), the oscillating nuclei “see” a broadband non-Lorentzian spectrum (Fig. 8), most of which is located outside the nuclear resonance in the region of relatively weak absorption and steep nuclear dispersion, similar to the case of the Gaussian photon (Fig. 3). It is this field that propagates through the absorber with small attenuation and slow velocity.

The small attenuation of the delayed part of the pulse corresponds to a decrease in the effective optical depth of the absorber from $T_{a} \approx 6$ to $T_{a}^{eff} \approx 0.4$, which also reduces the amplitude of dynamical beats. However, in contrast to the motionless absorber, the optical depth reduction of which also leads to elongation of the Sommerfeld-Brillouin precursor, a decrease in the effective optical thickness due to vibration of the same absorber does not change the Sommerfeld-Brillouin precursor.

With an increase in the absorber optical depth, the delay due to nuclear dispersion should also increase similar to the case of the Gaussian photon. However, in contrast to the Gaussian photon, a significant field of the overlapping Lorentzian sidebands is present in the vicinity of the resonant frequency (compare Fig. 3 and Fig. 8). The attenuation of this field due to resonant absorption increases with increasing optical depth, which shortens both the dynamical beats and the Sommerfeld-Brillouin precursor. These factors lead to a shift of the position of the second maximum in the photon waveform to an earlier moment (see Fig. 7 green line). Competition between the dynamical beats and the delay due to the steep nuclear dispersion defines the optimal optical depth, $T_{a}$, providing the maximum delay time. It is worth noting that the propagation delay and shape of the delayed part of the Lorentzian pulse in this case are also determined by the group velocity dispersion and non-uniform absorption over the field spectrum in the vibrating reference frame due to the long spectral wings of the Lorentz contour (Fig. 8). Their overlap is more pronounced than for the Gaussian spectrum (Fig. 3).

As shown in Figs. 6a and 7, the maximum delay for the Lorentzian photon in the ^57^Fe absorber of 144 ns exceeds the photon half-height duration of 97 ns. As can be seen from Fig. 7, this is the total delay of the delayed part of the Lorentzian photon, which can be represented as the sum of the delay due to the formation of a nuclear response of 76 ns (corresponding to the dip between the Sommerfeld-Brillouin precursor and the delayed part) and the delay of 68 ns due to steep dispersion of the formed nuclear response. This representation is rather qualitative, since the two processes are self-consistent and cannot be separated.

The propagation velocity of the 144 ns delayed part of the Lorentzian single-photon wave packet, estimated for the above stainless-steel foil (Fe_70_Cr_19_Ni_11_, 95% enriched with ^57^Fe, of the physical thickness 0.57 µm corresponding to $T_{a} = 6$), is $v \approx 4\;{{\text{m}} \mathord{\left/ {\vphantom {{\text{m}} {\text{s}}}} \right. \kern-\nulldelimiterspace} {\text{s}}}$ (see Fig. 6, green circles). Similar to the Gaussian photon, this velocity is not the smallest because of its dependence on the absorber optical depth. The minimum propagation velocity (Fig. 6b, orange circle), numerically estimated using Eqs. (35) and (33), is achieved at $T_{a} \approx 4$ (the corresponding physical thickness of the foil is $L \approx 0.38\,{\mu m}$), taking the value $v \approx 3\;{{\text{m}} \mathord{\left/ {\vphantom {{\text{m}} {\text{s}}}} \right. \kern-\nulldelimiterspace} {\text{s}}}$. It should be noted that the group velocity approximation in this case is invalid, since, both conditions Eqs. (41) and (42) are not satisfied.

It is worth noting that the single-photon waveform similar to waveform in Fig. 7 solid red line, including the Sommerfeld–Brillouin precursor and the second maximum corresponding to the delayed part, was observed earlier in the optical range where slowing down of the Lorentz-shape photon due to EIT was implemented^52^. Also, similar waveforms were observed in^39^ where slowing down of 14.4-keV photon in ^57^Fe-enriched compounds with a doublet upper state was realized.

Direct observation of the dispersive slowing-down of 14.4-keV Lorentzian photons in the vibrating absorber can be demonstrated via temporal filtration of the detected photons, which “cuts off” the Brillouin precursor from the waveform of the transmitted photon. Such a cutoff is usually used in synchrotron x-ray time-domain Mössbauer spectroscopy^32,33,38,45^. For parameter values of Fig. 7, this can be implemented if we start registering 14.4-keV photon in about 76 ns after detecting a 122-keV photon in the case of RMS or receiving a timing signal in the case of SMS. In Fig. 7 this corresponds to the photon waveform started at the vertical red dotted line. A dispersive delay of about 68 ns will correspond to the photon propagation velocity of about 8 m/s. Taking into account the delayed nuclear response to the stepwise front edge of the Lorentzian photon results in the effective propagation velocity of 3 m/s.

As shown in Fig. 7, the delayed fraction of the Lorentzian single-photon pulse contains 42% of the energy (the area under the red line to the right of the vertical red dotted line) of the incident pulse. This is significantly more than the transmitted energy in delayed optical pulses due to EIT (for example, 0.1% in a pulse having a velocity of 102 m/s^6^, up to 10% for 148 m/s^7^, and less than 15% for 32 m/s^2^).

## Slowing down Lorentz-squared photons

Synchrotron Mössbauer Source (SMS) available at ESRF^20,22^ and Spring-8 facility^23,24^ can operate in a single-line mode and produce single photons with various spectral forms. Let’s consider the case of ESRF when the SMS spectral line can be described by a superposition of two antiphase Lorentz lines Eq. (45) with HWHM $\gamma_{S}$ separated by $B$, which in the case $B \ll \gamma_{S}$ corresponds to the square of the Lorentz line^20^,

47 $$S_{in} \left( \delta \right) = \frac{1}{2\pi }\left[ {\frac{1}{{\gamma_{S} - i\left( {\delta + {B \mathord{\left/ {\vphantom {B 2}} \right. \kern-\nulldelimiterspace} 2}} \right)}} - \frac{1}{{\gamma_{S} - i\left( {\delta - {B \mathord{\left/ {\vphantom {B 2}} \right. \kern-\nulldelimiterspace} 2}} \right)}}} \right]\mathop \simeq \limits^{{B \ll \gamma_{S} }} \frac{1}{2\pi }\frac{iB}{{\left( {\gamma_{S} - i\delta } \right)^{2} }}.$$

The spectrum Eq. (47) has more rapidly falling wings than the Lorentz spectrum and corresponds to the smooth front of the photon waveform,

48 $$A\left( {t,0} \right) = 2i\theta (t)\sin \left( {{{Bt} \mathord{\left/ {\vphantom {{Bt} 2}} \right. \kern-\nulldelimiterspace} 2}} \right)e^{{ - \gamma_{S} t}} \mathop \simeq \limits^{{B \ll \gamma_{S} }} iB\theta (t)te^{{ - \gamma_{S} t}} .$$

**Figure 9:**
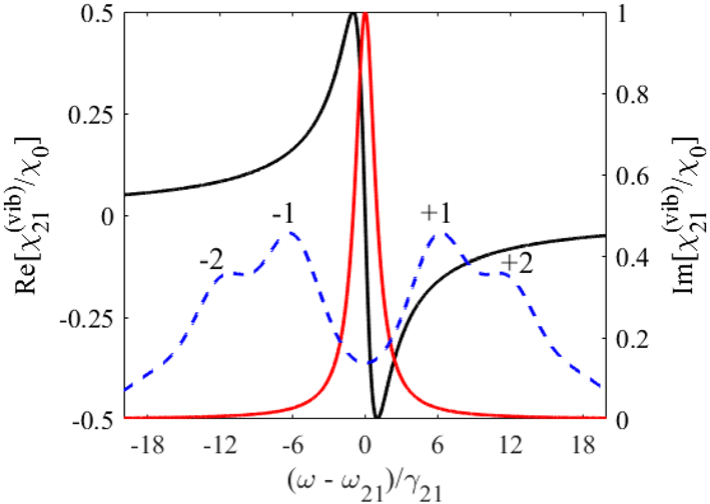
In the vibrating reference frame, real (black curve, left axis) and imaginary (red curve, right axis) parts of the resonant susceptibility Eq. (23) of the motionless absorber as well as the power spectral density of the resonant, $\omega_0=\omega_{21}$, spectrally broadened, $\Delta_{LS} = 3\gamma_{21}$ ($\gamma_{S} = 4.7\gamma_{21}$ and $B = 1.88\gamma_{21}$), incident wave packet with Lorentz-squared spectral profile Eq. (47) (blue dashed line) under ${\Omega \mathord{\left/ {\vphantom {\Omega {\gamma_{21} }}} \right. \kern-\nulldelimiterspace} {\gamma_{21} }} = 6$ and $p=p_1=2.4$.

We consider the experimentally implemented case, when $\gamma_{S} = 4.7\gamma_{21}$ and $B = 1.88\gamma_{21}$^20,23^. As follows from Eq. (47), the HWHM of the Lorentz-squared line is $\Delta_{LS} \approx 0.64\gamma_{S} \simeq 3\gamma_{21}$. Qualitatively, spectrum Eq. (47) is intermediate between Gaussian and Lorentz spectra. However, the width of the Lorentz-squared spectral contour is three times greater than the width of the Gaussian and Lorentzian spectra. Therefore, the group velocity dispersion over the field spectrum and the difference in absorption of the Fourier constituents of the field in the vibrating reference frame become more significant than in the case of Gaussian and Lorentzian photons (compare Figs. 3, 8 and 9). This can lengthen the pulse, change its shape, and shift the peak intensity. At the same time, the nuclear resonant absorption (red line in Fig. 9) affects a much smaller part of the field spectrum, which leads to the conservation of more energy in the pulse and reduces distortion in its shape.

**Figure 10:**
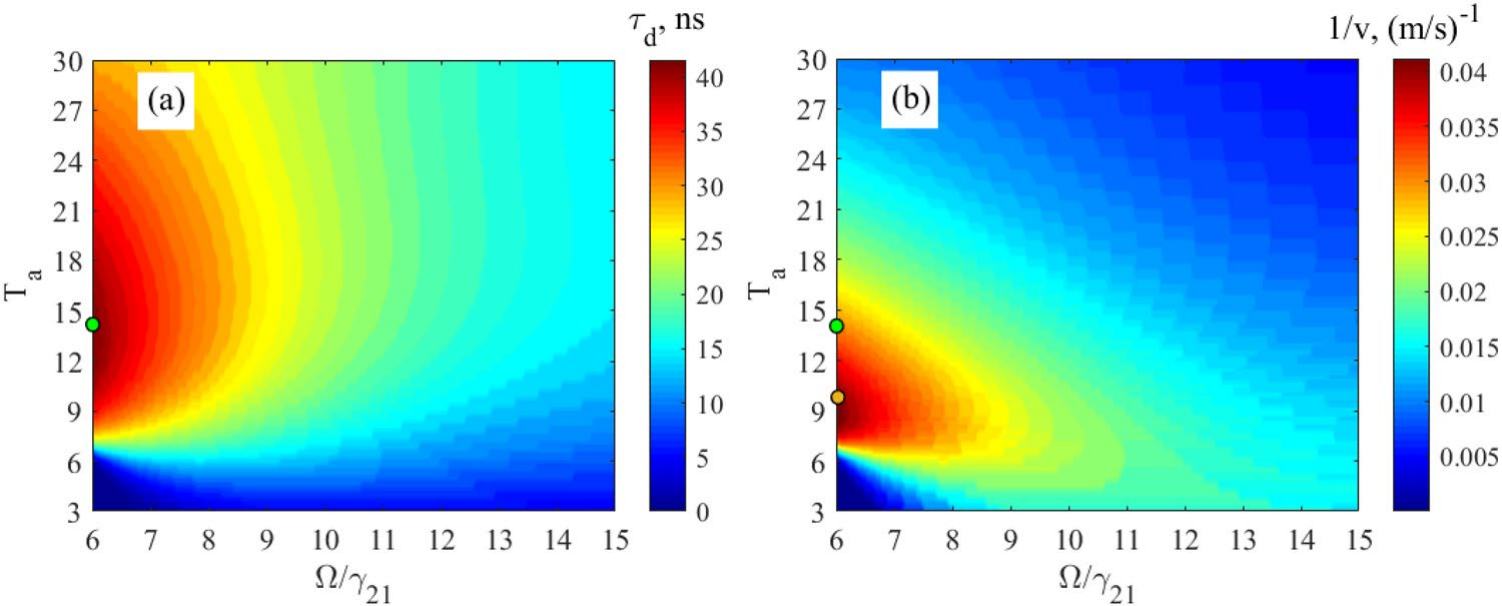
(**a**) The moment (in nanoseconds) of formation of the delayed pulse maximum, $\tau_{d}$**,** as a function of the absorber normalized vibration frequency, ${\Omega \mathord{\left/ {\vphantom {\Omega {\gamma_{21} }}} \right. \kern-\nulldelimiterspace} {\gamma_{21} }}$, and the resonant optical depth, $T_{a}$ (see Eq. (17)), plotted using numerical integration of Eq. (33), Eq. (47). The green circle marks the longest photon delay of 42 ns. (**b**) Dependence of the reciprocal propagation velocity, ${1 \mathord{\left/ {\vphantom {1 v}} \right. \kern-\nulldelimiterspace} v}$ in (m/s)^-1^, on the absorber vibration frequency, ${\Omega \mathord{\left/ {\vphantom {\Omega {\gamma_{21} }}} \right. \kern-\nulldelimiterspace} {\gamma_{21} }}$, and the optical depth, $T_{a}$, calculated according to Eq. (35) and panel (**a**). As above, the absorber is a stainless-steel foil Fe_70_Cr_19_Ni_11_, 95% enriched with ^57^Fe nuclide vibrating with amplitude $R_{1}$ in Eq. (14) corresponding to $p = p_{1}$ in Eq. (13). The green circle marks the propagation velocity of photons having the longest photon delay, which corresponds to the red waveform in Fig. 11 and equals 30 m/s. The orange circle marks the minimum propagation velocity of Lorentz-squared photons equal to 24 m/s.

**Figure 11:**
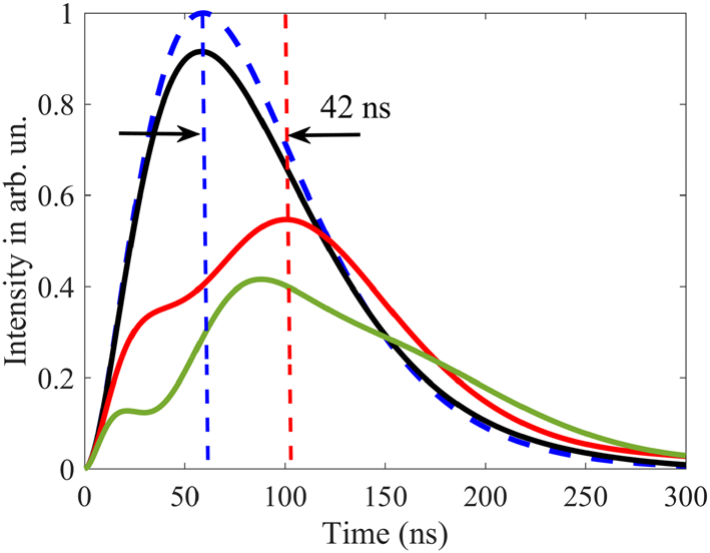
Normalized time dependence of the intensity of 14.4-keV Lorentz-squared single-photon wave packet Eq. (47) from ESRF SMS with a duration of 100 ns and peak intensity at the moment $t_{\max }^{{\left( {in} \right)}} = 59\;{\text{ns}}$. The blue dashed line corresponds to the incident wave packet, Eq. (47), under $B = 1.88\gamma_{21}$**,** $\gamma_{S} = 4.7\gamma_{21}$, ${{\gamma_{21} } \mathord{\left/ {\vphantom {{\gamma_{21} } {\left( {2\pi } \right)}}} \right. \kern-\nulldelimiterspace} {\left( {2\pi } \right)}} = 0.56\;{\text{MHz}}$. Other lines correspond to the photon waveform at the exit from the ^57^Fe absorber vibrating at the frequency ${\Omega \mathord{\left/ {\vphantom {\Omega {\gamma_{21} }}} \right. \kern-\nulldelimiterspace} {\gamma_{21} }} = 6$ with amplitude $R_{1} = 0.38\lambda$, Eq. (14), ($p_{1} = 2.4$, Eq. (13)), plotted using numerical integration of Eq. (33). The red line corresponds to the absorber optical depth $T_{a} = 13$; the green line corresponds to $T_{a} = 30$; the black solid line is plotted for $T_{a} = 1$.

In Fig. 10 we plot the delay and reciprocal propagation velocity of the Lorentz-squared single-photon wave packet from SMS as functions of the absorber vibration frequency and optical depth, using the definitions Eq. (34), Eq. (35), and numerical calculation of the intensity Eq. (33) Similar to the case of the Lorentzian photon, the optimal absorber vibration frequency and optical depth providing the longest propagation delay and lowest propagation velocity is located between two regions at the bottom and top of the panels, corresponding to relatively small pulse delay and high propagation velocity. In the region at the bottom, a small optical depth and relatively high vibration frequency of the absorber provide its relatively high transparency due to weak nuclear-field interaction (Fig. 11, black line). At the top, a high optical depth and relatively low vibration frequency of the absorber provide a relatively strong dynamical beats in the photon waveform, which distort the pulse shape and shift its peak to earlier times similar to the Lorentzian photon (Fig. 11, green line). Similar to the Lorentzian photon, for any given vibration frequency, the competition between dynamical beats and nuclear dispersion leads to some optimal value of the absorber optical depth, which provides the longest propagation delay and the slowest propagation velocity. However, the Lorentz-squared spectrum has significantly smaller wings than the Lorentz spectrum, which leads to much weaker dynamical beats and less distortion in the transmitted waveform, including the absence of the Sommerfeld-Brillouin precursor. Similar to the Gaussian and Lorentzian photons, the optimal absorber vibration frequency for both the longest pulse delay and slowest propagation velocity satisfies the relation Eq. (44) that becomes ${\Omega \mathord{\left/ {\vphantom {\Omega {\gamma_{21} }}} \right. \kern-\nulldelimiterspace} {\gamma_{21} }} \approx 6$. The optical depth of the absorber for the longest pulse delay of $\tau_{d} \approx 42\;{\text{ns}}$ is $T_{a} \approx 13$ (see Fig. 10a, green circle). In this case, the waveform of the delayed Lorentz-squared photon is plotted in Fig. 11, red line.

The propagation velocity of the Lorentz-squared single-photon pulse in the case of ${\Omega \mathord{\left/ {\vphantom {\Omega {\gamma_{21} }}} \right. \kern-\nulldelimiterspace} {\gamma_{21} }} \approx 6$ and $T_{a} \approx 13$ is equal to $v \approx 30\;{{\text{m}} \mathord{\left/ {\vphantom {{\text{m}} {\text{s}}}} \right. \kern-\nulldelimiterspace} {\text{s}}}$ (see also Fig. 10b, green circle). Similar to the case of the Lorentzian photon, it is not the slowest propagation velocity due to its dependence on the absorber optical depth. As can be seen in Fig. 11b, orange circle, the minimum propagation velocity of the Lorentz-squared photon is $v \approx 24\;{{\text{m}} \mathord{\left/ {\vphantom {{\text{m}} {\text{s}}}} \right. \kern-\nulldelimiterspace} {\text{s}}}$. It is achieved at ${\Omega \mathord{\left/ {\vphantom {\Omega {\gamma_{21} }}} \right. \kern-\nulldelimiterspace} {\gamma_{21} }} \approx 6$ and $T_{a} \approx 9$ (the corresponding physical thickness of the foil is $L \approx 0.84\,{\mu m}$). As in the case of the Lorentzian photon, the group velocity approximation in this case is invalid, since both conditions Eqs. (41) and (42) are not satisfied.

As shown in Fig. 11, the delayed Lorentz-squared single-photon pulse contains 73% of the energy (the area under the red line) of the incident pulse.

## Conclusion

In this paper, we theoretically studied the effect of slowing down the propagation velocity of radiation in a medium under conditions of acoustically induced transparency (AIT) in the case of 14.4-keV photons and a ^57^Fe recoilless nuclear absorber. We have shown that the main mechanism for slowing down the propagation velocity of a single-photon wave packet is the steep dispersion of the resonant nuclear transition within the transparency spectral window created at the position of the nuclear resonance (see Fig. 2). This feature of AIT is similar to the effects of electromagnetically induced transparency (EIT) and Autler-Townes splitting (ATS). In contrast to EIT and ATS, where the transparency window is created by quite intense radiation in a three- or multilevel system, AIT-spectral window is induced in a two-level system by the collective oscillations of nuclei with the same amplitude as a result of a piston-like absorber vibration. We considered three types of the 14.4-keV single-photon wave packets, namely Gaussian, Lorentzian and Lorentz-squared. Analysis of the Gaussian wave packet gives a clear physical picture of photon deceleration via AIT. Analysis of the Lorentzian and Lorentz-squared 14.4-keV single-photon wave packets provides a theoretical background for observation and study of slow 14.4-keV photons at the existing experimental conditions, using the ^57^Co radioactive Mössbauer source (RMS) and synchrotron Mössbauer Sources (SMS) available at ESRF^20,22^ and Spring-8 facility^23,24^.

We have found optimal experimental conditions under which 14.4-keV photons can be strongly slowed down and delayed in a ^57^Fe-enriched stainless-steel foil at room temperature with relatively small losses in the intensity of a single-photon wave packet. In particular, a significant portion containing more than 40% of the energy of 97-ns duration single-photon wave packet from RMS can be slowed down to 3 m/s and delayed by 144 ns. A reduction in the propagation velocity to 24 m/s and a delay of 42 ns can be achieved for 100-ns length photons from SMS with a conservation of more than 70% of the energy. These values can be improved via higher doping of the stainless-steel with ^57^Fe nuclei (98% doping was used in^54^) as well as via increase of the Lamb-Mössbauer factor by decreasing the absorber temperature (at room temperature $f_{a} < 0.8$ whereas $f_{a} > 0.9$ at temperature below 50 K) or by using a host material with a harder crystal lattice (for ^57^Fe implanted into a diamond crystal, $f_{a} \approx 0.94$ at 295 K^55^). We have also shown that the propagation velocity can be widely controlled by changing the absorber vibration frequency.

The propagation velocities of hard x-ray and γ-ray photons on the order of units to several tens of meters per second are much lower than the propagation velocities currently achieved in this frequency range. They are comparable to the currently achieved slowest velocities of visible light. However, they could be attained in much thinner (sub-micrometer-thick) absorber. Slowing down hard x-ray and γ-ray photons under the AIT condition can open novel prospects for manipulating high-energy photons similar to EIT slow light in optical range.
